# Genomic Characterization and Antimicrobial Susceptibility of Dromedary-Associated *Staphylococcaceae* from the Horn of Africa

**DOI:** 10.1128/aem.01146-22

**Published:** 2022-10-13

**Authors:** Hatice Akarsu, Anne Liljander, Mario Younan, Isabelle Brodard, Gudrun Overesch, Ilona Glücks, Fabien Labroussaa, Peter Kuhnert, Vincent Perreten, Stefan Monecke, Jan Felix Drexler, Victor Max Corman, Laurent Falquet, Joerg Jores

**Affiliations:** a Institute of Veterinary Bacteriology, University of Bern, Bern, Switzerland; b SIB Swiss Institute of Bioinformatics, Switzerland; c International Livestock Research Institute, Nairobi, Kenya; d Food and Agriculture Organization of the UN (FAO), UN Cross-Border Hub for NW Syria, Gaziantep, Turkey; e Multidisciplinary Center for Infectious Diseases, University of Bern, Bern, Switzerland; f Friedrich Schiller University Jena, Institute of Physical Chemistry, Jena, Germany; g Institute of Virology, Charité, Universitätsmedizin Berlin, Freie Universität Berlin, Humboldt-Universität, Berlin Institute of Health (BIH), Berlin, Germany; h Biochemistry Unit, University of Fribourg, Fribourg, Switzerland; Centers for Disease Control and Prevention

**Keywords:** *Staphylococcus*, *Mammaliicoccus*, camel, phage, resistance, virulence

## Abstract

Members of the *Staphylococcaceae* family, particularly those of the genus Staphylococcus, encompass important human and animal pathogens. We collected and characterized *Staphylococcaceae* strains from apparently healthy and diseased camels (*n* = 84) and cattle (*n* = 7) in Somalia and Kenya. We phenotypically characterized the strains, including their antimicrobial inhibitory concentrations. Then, we sequenced their genomes using long-read sequencing, closed their genomes, and subsequently compared and mapped their virulence- and resistance-associated gene pools. Genome-based phylogenetics revealed 13 known *Staphylococcaceae* and at least two novel species. East African strains of different species encompassed novel sequence types and phylogenetically distant clades. About one-third of the strains had non-wild-type MICs. They were resistant to at least one of the following antimicrobials: tetracycline, benzylpenicillin, oxacillin, erythromycin, clindamycin, trimethoprim, gentamicin, or streptomycin, encoded by *tet*(K), *blaZ*/*bla_ARL_*, *mecA*/*mecA1*, *msrA*/*mphC*, *salA*, *dfrG*, *aacA-aphD*, and *str*, respectively. We identified the first methicillin- and multidrug-resistant camel S. epidermidis strain of sequence type (ST) 1136 in East Africa. The pool of virulence-encoding genes was largest in the S. aureus strains, as expected, although other rather commensal strains contained distinct virulence-encoding genes. We identified toxin-antitoxin (TA) systems such as the *hicA/hicB* and *abiEii/abiEi* families, reported here for the first time for certain species of *Staphylococcaceae*. All strains contained at least one intact prophage sequence, mainly belonging to the *Siphoviridae* family. We pinpointed potential horizontal gene transfers between camel and cattle strains and also across distinct *Staphylococcaceae* clades and species.

**IMPORTANCE** Camels are a high value and crucial livestock species in arid and semiarid regions of Africa and gain importance giving the impact of climate change on traditional livestock species. Our current knowledge with respect to *Staphylococcaceae* infecting camels is very limited compared to that for other livestock species. Better knowledge will foster the development of specific diagnostic assays, guide promising antimicrobial treatment options, and inform about potential zoonotic risks. We characterized 84 *Staphylococcaceae* strains isolated from camels with respect to their antimicrobial resistance and virulence traits. We detected potentially novel Staphylococcus species, resistances to different classes of antimicrobials, and the first camel multidrug-resistant S. epidermidis strain of sequence type 1136.

## INTRODUCTION

In many semiarid and arid regions of Africa, keeping dromedary camels is the most sustainable livestock enterprise. Due to climate change and desertification, numbers of cattle and small ruminants are decreasing in these regions, while camels are increasing in number and likely to play an even more significant role for human livelihood in the future (1–3). For the people living in these harsh, dry areas, camels play a pivotal role in survival as important sources of animal protein, means of transportation, symbols of cultural status, and financial assets (3, 4). Camel-keeping societies live in very close contact with their animals, fostering exchange of microorganisms. Camel milk is traditionally consumed raw, which poses a risk of acquiring infections with zoonotic pathogens (4). Camels, which can provide milk until their late twenties, are the sub-Saharan livestock species with the highest average value per animal, and their health status impacts not only their productivity but also their net value. In the past few years, camels of East Africa have received scientific attention with respect to zoonotic viruses such as coronaviruses (5, 6) and hepatitis E virus (7). Camels have been shown to harbor and be affected by major bacterial pathogens such as Streptococcus agalactiae (8) and Staphylococcus aureus (9, 10).

Members of the family *Staphylococcaceae* currently include 10 validly published genera encompassing 122 validly published species (https://lpsn.dsmz.de/) (11). They have a genome size of 2 to 3 Mb with a GC content of 31% to 39%. Staphylococci are traditionally classified into coagulase-positive (CoPS), such as S. aureus, and coagulase-negative staphylococci (CoNS). The human and veterinary relevance of CoNS is also being increasingly investigated. Several CoNS have shown potential pathogenicity using S. aureus-like virulence traits or alternative mechanisms (12), especially for host immune response escape, by developing adherence to extracellular matrices or biomaterials and by forming protective biofilms. CoNS commensal strains can also act as reservoirs of antimicrobial resistance-encoding genes (ARG), as shown, for example, with Mammaliicoccus sciuri, Staphylococcus simulans, S. chromogenes, and S. pasteuri in livestock-associated environments (13). Only a limited number of studies have investigated the distribution of virulence factors and resistance-encoding genes in S. aureus strains isolated from dromedary camels (9, 10, 14). Currently, there is a paucity of phenotypical and genotypical data on dromedary camel-derived *Staphylococcaceae* strains, impairing the development of specific diagnostic tools and assessment of their zoonotic risk.

In this study, we phenotypically and genotypically characterized 84 camel-derived and 7 cattle-derived *Staphylococcaceae* strains which were collected from diseased and apparently healthy animals using convenience sampling. First, we tested their biochemical properties with respect to utilization of different energy sources and antimicrobial resistance patterns. This was followed by PacBio sequencing, leading to high-quality genomes which were subjected to comparative genomics, including defining their pan and core genomes, phylogenetic position, methylation profile diversity and associated candidate methyltransferases (MTase), presence of resistance genes, virulence trait-encoding genes, and toxin-antitoxin (TA) systems. We detected a pool of virulence factor- and resistance-encoding genes circulating in the commensal and opportunistic pathogens on top of their horizontal gene transfer (HGT) genetic signatures.

## RESULTS

### Initial phenotypic species assignment of the *Staphylococcaceae* strains revealed 13 known species.

**FIG 1:**
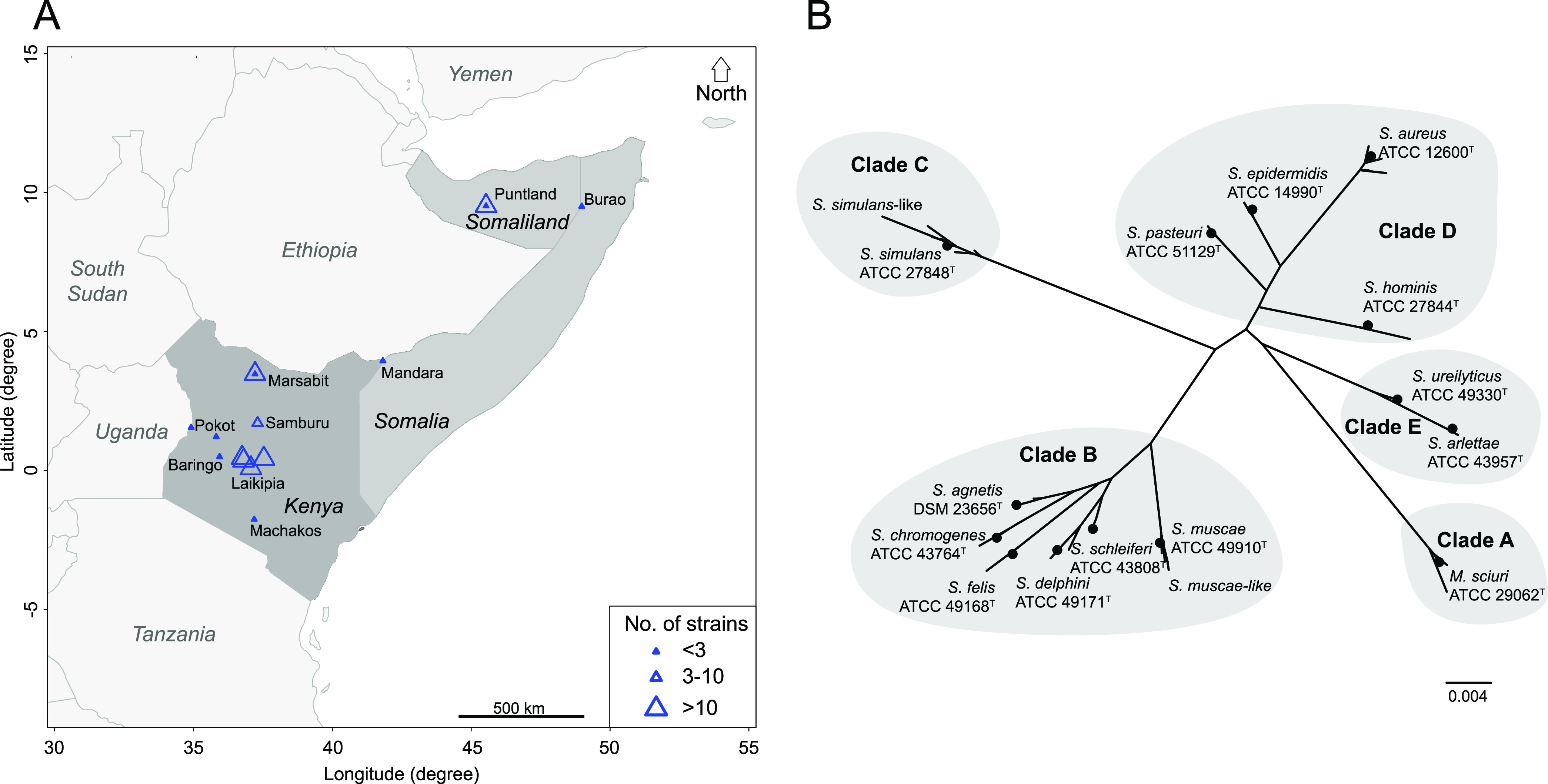
Geographic origin of strains and species investigated. (A) Map of the Horn of Africa showing the regions where the *Staphylococcaceae* strains were sampled. Triangle size reflects the number of strains collected in a given area. (B) Phylogenetic analysis of *Staphylococcaceae* (*n* = 91): unrooted tree based on 16S rRNA gene (*rrn*). 16S rRNA gene sequences were extracted from the complete genomes and aligned with MUSCLE. The unrooted phylogenetic tree was built with PhyML using the GTR substitution model.

For this study, 91 strains were collected in the Horn of Africa from different regions in Kenya and Somalia (Fig. 1A) using convenience sampling. These 91 strains included 7 isolated from cattle kept in the vicinity of camels to determine whether they shared common properties with the 84 strains isolated from camels. The metadata associated with all strains of this study are listed in Data Set S1 in the supplemental material. The initial species designation was based on matrix-assisted laser desorption ionization–time of flight mass spectrometry (MALDI-TOF MS) analysis in parallel with biochemical phenotyping on a VITEK 2 Gram-Positive (GP) card (Data Set S1). Phenotypic characterization revealed a minimum of 12 known Staphylococcus species and Mammaliicoccus sciuri (11) (Fig. 1B). Fifty strains (~65% of the total) were derived from clinical samples and 24 from apparently healthy animals, and the remaining strains were isolated from animals for which the health status was unknown (Data Set S1). Forty-four strains had a beta-hemolytic phenotype (Table 1). All strains of the species S. arlettae and S. ureilyticus were isolated from apparently healthy animals, while all strains from S. agnetis, S. hominis, S. pasteuri, and S. schleiferi were isolated from infection sites of animals with clinical disease. All remaining strains of different species were isolated from animals which were either diseased, apparently healthy, or had unknown clinical status (Data Set S1).

**TABLE 1:** East African *Staphylococcaceae* strains used in this study^*a*^

Species (no. of bovine strains)	Total strains/no of strains isolated from clinical specimens	Nature of clinical specimen (no of strains)	Non-wild-type AMR strains (*n*)	β-Hemolytic strains (*n*)	ST profiles (no of strains)
Mammaliicoccus sciuri	10/0	NA	3	0	59 (1), 67 (1), 202 (5), 203 (1), 204 (1), 205 (1)
Staphylococcus agnetis	6/6	WI (2); SW (1); RI (1); II (2)	1	0	NA
S. arlettae	2/0		0	0	NA
S. aureus (4)	33/24	WI (10); RI (3); II (7); EI (3); LAP (1)	9	33	30 (8), 1765 (1), 2073 (5), 2957 (4), 3573 (2), 3575 (1), 3576 (1), 3581 (3), 3582 (1), 3583 (3), 3602 (1), 3667 (1), 7619 (1), 7620 (1)
S. chromogenes (2)	2/0	NA	1	0	128 (1), 129 (1)
S. delphini	6/1	II (1)	1	6	NA
S. epidermidis (1)	5/3	II (3)	5	0	6 (1), 252 (2), 441 (1), 1136 (1)
S. felis	1/0	0	0	1	NA
S. hominis	1/1	LAP (1)	1	0	75 (1)
S. muscae*-*like	7/0	0	0	0	NA
S. pasteuri	1/1	II (1)	1	0	NA
S. schleiferi	4/4	WI (2); EI (1); RI (1)	0	4	NA
S. simulans	11/9	II (6);WI (3)	1	0	NA
S. simulans*-*like	1/1	WI (1)	0	0	NA
S. ureilyticus	1/0	0	1	0	NA
Complete dataset	91/50	50	24	44	

a Strains were obtained from camel (*n* = 84) and cattle (*n* = 7) hosts. NA, not applicable; AMR, antimicrobial-resistant; II, intramammary infection (milk); RI, respiratory infection (nasal swab); WI, wound infection (swab); EI, ear infection (swab); LAP, lymphadenopathy (swab); SW, sick weaner (nasal swab).

### MICs against different antimicrobials.

**FIG 2:**
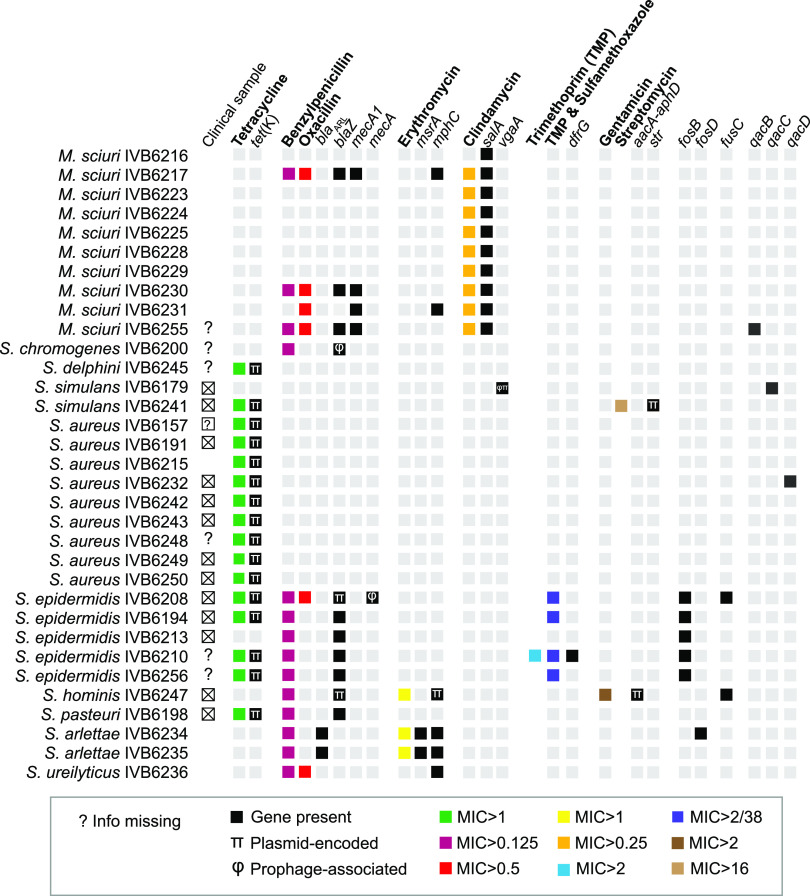
Phenotypic and genetic features of the East African *Staphylococcaceae* strains (*n* = 91) related to resistance against antimicrobials and disinfectants. Phenotypic antimicrobial susceptibility testing was done by MIC (μg/mL) testing. Antimicrobials are indicated in bold. Resistance genes associated with prophage sequence were flagged with the symbol φ; plasmid-located resistance genes were labeled with the symbol π. Boxes in gray represent wild-type phenotypes or the absence of genes.

We tested MICs against different antimicrobials to obtain initial data on the trends of resistance development in bacteria colonizing the extensively reared dromedary camels in East Africa and to later pinpoint the cognate resistance genes. All strains were first screened using the VITEK 2 AST-GP80 card to identify strains with non-wild-type MIC distributions. After identifying strains with non-wild-type MIC distributions, we determined their MICs against different antimicrobials using the Thermo Fisher Scientific SENSITITRE system (Data Set S1). Overall, 33 strains showed non-wild-type MIC distributions for at least one antimicrobial. More specifically, non-wild-type MIC distributions were observed against (i) tetracycline (MIC > 1 μg/mL) for 9/33 S. aureus, 4/5 S. epidermidis, 1/11 *S. simulans*, 1/6 S. delphini, and 1/1 *S. pasteuri* strain; (ii) benzylpenicillin (MIC > 0.125 μg/mL) for 5/5 S. epidermidis, 3/10 *M. sciuri*, 2/2 *S. arlettae*, 1/2 *S. chromogenes*, 1/1 S. hominis, 1/1 *S. pasteuri*, and 1/1 *S. ureilyticus* strain; (iii) oxacillin (MIC > 0.5 μg/mL) for 4/10 *M. sciuri*, 1/5 S. epidermidis, and 1/1 *S. ureilyticus* strain; (iv) erythromycin (MIC > 1 μg/mL) for 2/2 *S. arlettae* and 1/1 S. hominis strain; (v) clindamycin (MIC > 0.25 μg/mL) for 9/10 *M. sciuri* strains; (vi) trimethoprim (MIC > 2 μg/mL) for one S. epidermidis strain; (vii) trimethoprim-sulfamethoxazole (MIC > 2/38 μg/mL) for 4/5 S. epidermidis strains; (viii) gentamicin (MIC > 2 μg/mL) for 1/1 S. hominis strain; and (ix) streptomycin (MIC > 16 μg/mL) for 1 *S. simulans* strain tested (Data Set S1, Fig. 2). Non-wild-type phenotypes against clindamycin, erythromycin, gentamicin, and streptomycin were only present in the Kenyan strains.

### Genome assemblies.

The 91 strains were sequenced with PacBio long-reads, and the genomes were assembled with two distinct methods for robustness: namely, Flye (15) and SMRTLink. All chromosomes used in the study were circularized, with high coverage values ranging from 234× to 1,250×; chromosome length ranged from 2,181,984 to 2,923,078 bp, with GC content values varying from 31.61% to 38.36% (Data Set S2). Forty-eight out of 91 strains had one or more circularized plasmids.

### Phylogeny based on 16S rRNA gene sequences.

Next, we wanted to confirm the MALDI-TOF MS-derived species designation with the species designation based on *rrn* sequences. All chromosomes had five 16S rRNA (*rrn*) operons. We built an unrooted phylotree using the full 16S rRNA gene sequence derived from one *rrn* operon per strain as well as from the type strains of the species detected (Fig. 1B). Interestingly, we noticed extra branches in clades B and C, named *S. simulans*-like and *S. muscae*-like (Fig. 1B). These branches could represent novel species, and they were investigated next.

### Average nucleotide identity analysis confirms known species and indicates novel undescribed species.

**FIG 3:**
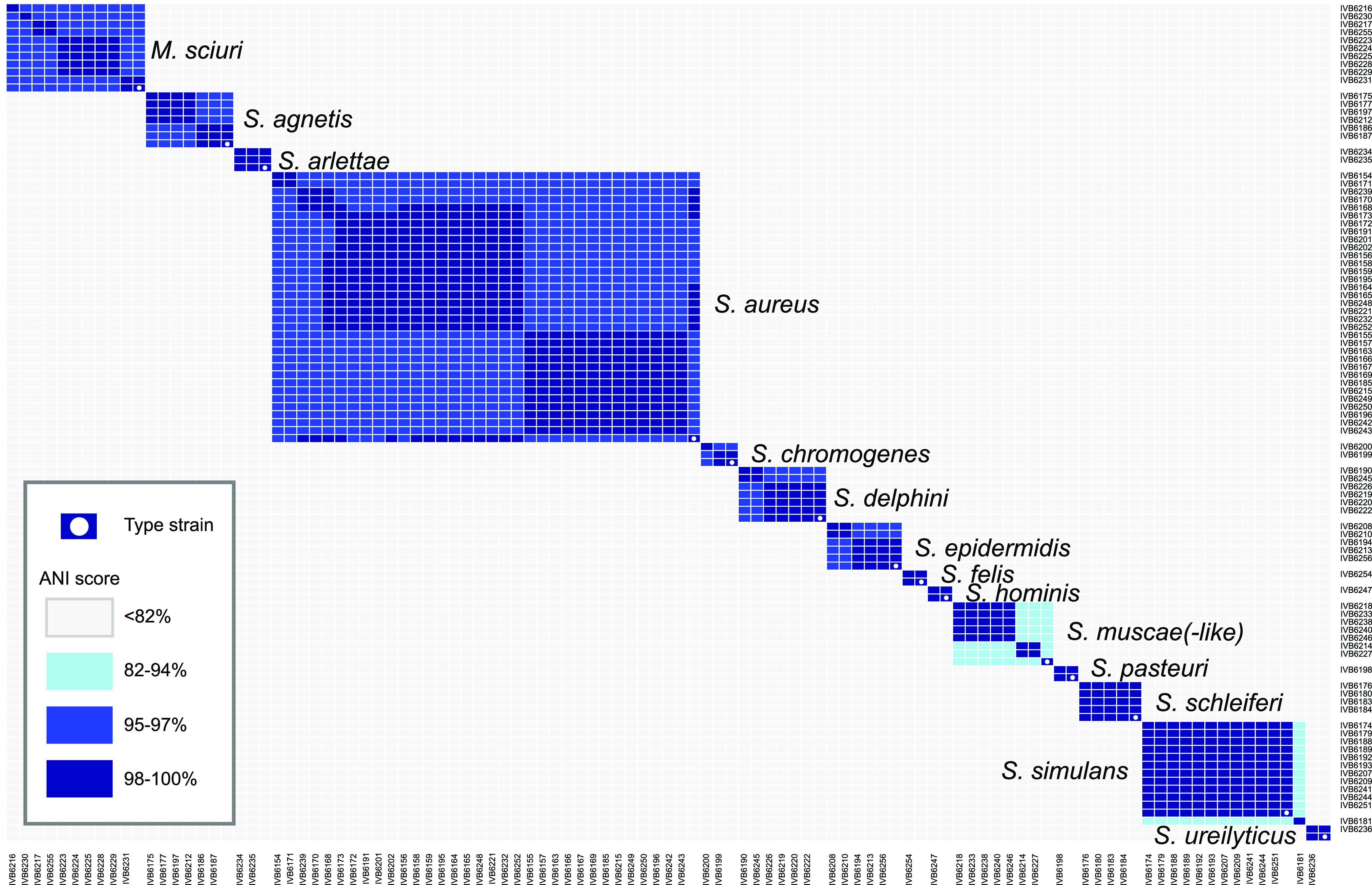
Relatedness of the strains investigated, depicted by a heatmap based on average nucleotide identity (ANI) scores. The software FastANI was used to generate the values and the R heatmap.2 method was used to plot the summary heatmap. An ANI score of ≥95% defines strains which belong to the same species.

The whole-genome-based all-against-all average nucleotide identity (ANI) comparisons with FastANI (16) and clustering (Fig. 3) were overall coherent with the MALDI TOF MS results described above (Data Set S1). The highest ANI score for strain IVB6181, labeled as *S. simulans*-like, was 83.9% when paired with *S. simulans* IVB6174, far below the taxonomic species threshold of ≥95% for ANI data (17). Species identification performed on the Type Strain Genome Server TYGS (18) did not attribute any known species to IVB6181. The ANI heatmap (Fig. 3) indicated 7 strains belonging to undescribed species which were phylogenetically related to *S. muscae*, here referred to as *S. muscae*-like strains. These *S. muscae*-like strains were divided into two groups. One group consisted of two closely related strains, IVB6214 and IVB6217 (>99.7% ANI score for this pair), and another group was formed by 5 other *S. muscae*-like strains (ANI scores ranging from 99.3% to 99.8% in pairwise comparisons of the 5 strains to each other). This suggested that the 7 *S. muscae*-like strains represent new distinct species. Again, our TYGS analysis did not result in any clear species designation of the 7 *S. muscae*-like strains. Future studies will shed light on the taxonomic positions of the *S. simulans*-like and *S. muscae*-like strains.

### Phylogenomic analysis using core genome data identified camel-specific lineages.

Next, we wanted to investigate the phylogenetic position of the East African strains within the species, and we built phylogenetic trees based on core genome data. To determine the core genomes, we proceeded with comparative genomics of *Staphylococcaceae* strains against the genomes available in GenBank (Data Set S2). A quick glance at the number of complete genomes (GenBank, accessed May 2022, see also Data Set S2) showed how our data set compellingly expanded the number of complete genomes for *Staphylococcaceae* other than S. aureus and S. epidermidis. None of the species other than S. aureus and S. epidermidis had any complete genomes of strains isolated from camels, which underlines the novelty of the data set generated. We collected the genome sequences available for each of the relevant species (Data Set S2) and analyzed the phylogenetic relationship of our strains with the currently existing genomes in the NCBI database. We ran pan-core clustering based on the genetic content of each genome to obtain a core genome and built the corresponding phylotrees (Fig. S1). The strains in this study were often scattered across the corresponding phylotrees. We also observed that the camel strains tended to cluster together into monophyletic nodes. For instance, the two camel-derived *S. agnetis* strains (IVB6186 and IVB6187) colocalized in the poultry group (19), while the remaining camel *S. agnetis* strains—namely, IVB6175, IVB6177, IVB6197, and IVB6212—formed a distinct branch within the non-poultry subset of the tree (Fig. S1B). Interestingly, the S. aureus core genome phylotree showed that the four cattle-derived S. aureus strains (IVB6172, IVB6191, IVB6201, and IVB6202) clustered together in a separate subgroup of the core genome phylotree (Fig. S1C).

### Multilocus sequence typing reveals camel-specific sequence types.

We used multilocus sequence typing (MLST) profiling for S. aureus, S. epidermidis, *S. chromogenes*, S. hominis, and *M. sciuri* because these species had established MLST schemes (Data Set S2). New alleles and sequence types (STs) were added to the databases. Next, we built minimum spanning trees (MSTs) by combining the ST profiles from these data sets with those available in PubMLST to view the positioning of our strains in relation to the PubMLST-deposited strains, attributing clonal complex (CC) when possible (Fig. S1O to S).

The S. aureus strains analyzed clustered within CCs known to harbor clinical human strains and within STs specific for livestock. For instance, camel strains belonged to CCs which also contained human strains (CC30, CC93, and CC121), as well as to livestock CCs such as CC1765 which harbored only camel strains (Fig. S1O). These data are in line with the phylogenies based on core genome data.

The 5 S. epidermidis strains (Fig. S1P) belonged to 4 STs. Only ST-1136 was camel-specific and novel; ST-441 was cattle-specific. ST-6 and ST-251 contained the camel strains of this study and the human strains.

The 10 *M. sciuri* strains (Fig. S1Q) belonged to 6 STs, while 5 of the latter were represented by only 1 strain and 5 strains belonged to ST-202. Only ST-67 and ST-59 contained strains isolated from spiders and cattle, respectively.

The only S. hominis strain of this study represented ST-75 (Fig. S1R). The 2 bovine *S. chromogenes* strains of this study belonged to ST-128 and ST-129 (Fig. S1S).

### Detection of antimicrobial resistance genes and genes encoding resistance to disinfectants.

Next, we investigated the presence of antimicrobial resistance genes after confirming non-wild-type strains with respect to the MIC against a variety of antimicrobials. We linked the phenotypic characterization described above to the ARG pool with an *in silico* screen using ResFinder (20). Afterwards, we matched the phenotypic data with the genotypic data (Fig. 2). Tetracycline resistance was associated with the presence of the tetracycline efflux gene *tet*(K). Beta-lactam resistance was associated with *bla_ARL_* and *blaZ*, both encoding beta-lactamases. Oxacillin resistance was associated with *mecA1* and *mecA*, encoding a penicillin-binding protein 2A. Erythromycin resistance was associated with *msrA* or *mphC*, encoding an ABC-F subfamily ribosomal protection protein and a macrolide phosphotransferase, respectively. Clindamycin resistance was associated with *salA*, encoding an ABC-F subfamily protein. Trimethoprim resistance was linked to *dfrG*, encoding dihydrofolate reductase. Gentamicin resistance was linked to *aacA-aphD*, encoding an aminoglycoside acetyltransferase. Finally, streptomycin resistance was associated with *str*, encoding aminoglycoside 6-adenylyltransferase. However, the limited phenotypic data obtained did not allow us to match genotypic data for the genes *fosB*, *fosD*, and *fusC*.

### Virulome profiling highlights the presence of many virulence traits in *S. aureus* strains, to a lesser extent in non-*aureus Staphylococcaceae*.

**FIG 4:**
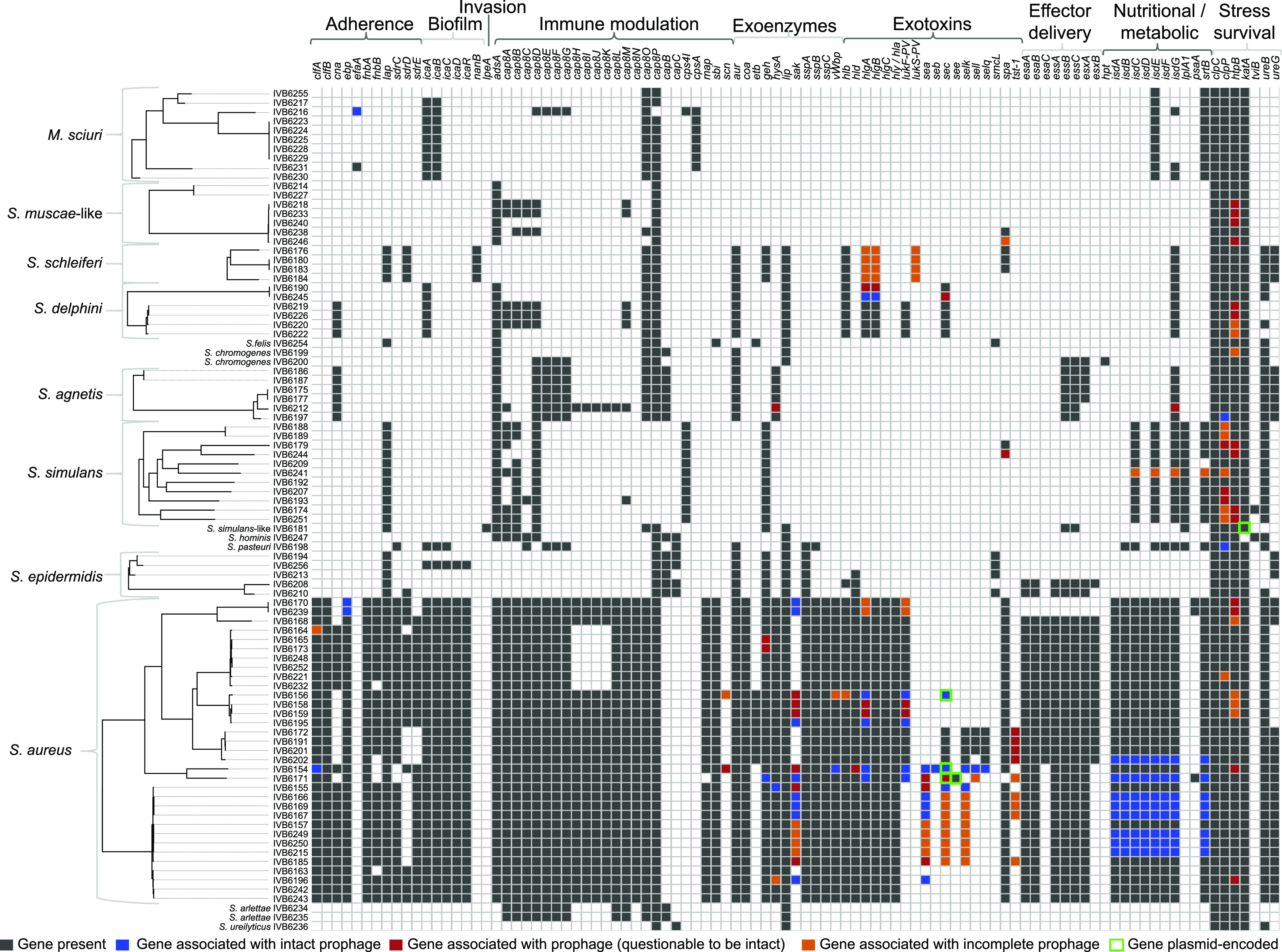
Virulence attributes determined by *in silico* screening of *Staphyloccaceae* strains (*n* = 91). The phylotrees on the left were constructed using core genome data and the virulence traits depicted were selected based on the virulence factor (VF) database. S. aureus clearly has the most virulence factor-encoding genes in contrast to the other species. Nevertheless, all species contain at least six VF-encoding genes. Only a fraction of the VF-encoding genes is part of the mobilome consisting of bacteriophages and plasmids (see color code).

To shed light on the virulence factor (VF)-encoding genes present in the different species investigated, we performed an *in silico* screen for the presence of VF-encoding genes (Fig. 4). More than 80% of the candidate VF-encoding genes had a percentage of DNA identity and coverage above 70%. Overall, 11% of the putative VF-encoding genes hinted toward potentially shorter variants or truncations.

The clustering of the VFs highlighted a subgroup of S. aureus strains (IVB6249, IVB6215, IVB6250, IVB6202, IVB6167, IVB6166, IVB6169, and IVB6171) presenting VFs from the exotoxin category; in particular, staphylococcal superantigens, including enterotoxin genes *sea*, *sec*, *selk*, and the toxic-shock-syndrome-toxin 1 gene *tst-1*. The latter VF is known to encode a potent toxin which causes toxic shock syndrome. The same strains also possessed *isd* factors (*isdA-G*) involved in iron uptake from the host. In this study, *tst-1* was found in all four bovine S. aureus strains (IVB6191, IVB6172, IVB6201, and IVB6202) and in five S. aureus camel strains (IVB6167, IVB6185, IVB6166, IVB6169, and IVB6171). The *scn* gene, involved in the inhibition of the host primary immune response, was present in two camel S. aureus strains (IVB6154 and IVB6156). Finally, the clumping factor A, encoded by the *clfA* gene, was detected in all S. aureus strains in the data set. Among the genes related to adherence, *ebp*, coding for the elastin-binding protein, was present in all 33 S. aureus strains. Similarly, the gene *vwb*—encoding the secreted von Willebrand factor-binding protein—was present in all strains. All S. aureus strains were positive for the alpha pore-forming toxin and bi-component pore-forming toxins in the *in silico* screen (Fig. 4). A closer look (Data Set S3) at the bi-component toxins revealed 2 to 4 different bi-component toxin pairs (21, 22) per strain (Fig. S2A to B). The four *S. schleiferi* strains (Fig. S2C to E) and the S. delphini strains (Fig. S2F to H) were also positive for bi-component toxins.

**FIG 5:**
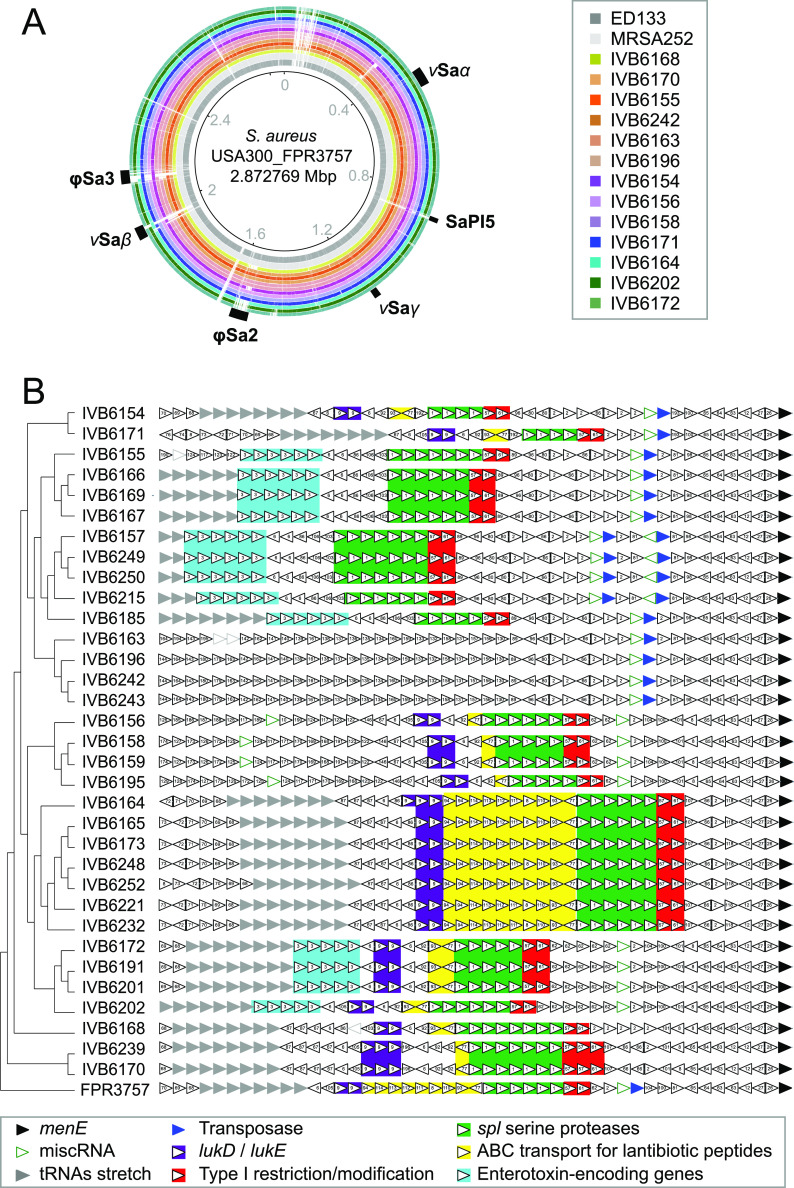
Examples of VF-encoding genes and signatures releated to horizontal gene transfer (HGT). (A) Pathogenicity islands (PAIs) map for selected S. aureus strains. The S. aureus strain USA300_FPR3757 (GenBank no. NC_007793) was used as a reference for mapping its annotated PAIs, as provided in the Pathogenicity Island Database (PAIDB). (B) Zoom on the *v*Sa*β* PAI region. Genomic neighborhood comparison of the PAI locus across the reference S. aureus strain (USA300_FPR3757) and the 33 S. aureus strains from this data set. A core genome alignment (based on 1,982 core genes) was obtained with Roary and MAFFT, followed by a phylotree reconstruction using IQTREE and PhyML. S. aureus USA300_FPR3757 was used as the outgroup. This core genome phylotree was then plotted against the gene neighborhood analysis derived from the FlaGs tool. Details (e.g., virulence factors and resistance genes) of the PAI region are highlighted as colored boxes.

Several pathogenicity islands (PAIs) have been reported for S. aureus. We investigated the presence of the PAIs *v*Sa*α*, SaPi5, *v*Sa*γ*, φSa2, *v*Sa*β*, and φSa3 in the representative S. aureus strains from our data set (Fig. 5A). We retrieved the PAI sequences from the reference S. aureus USA300_FPR3757 via the Pathogenicity Island Database (PAI DB) (23) and analyzed our subset of strains using BRIG (BLAST Ring Image Generator) (24) (Fig. 5A). We detected the six PAIs at different levels of conservation across the S. aureus strains in this study (Fig. 5B). The PAIs vSaα and vSaγ appeared more conserved across the strains, while the PAIs SaPi5, φSa2, vSaβ, and φSa3 showed more heterogeneity between different S. aureus strains (Fig. 5B).

### The intra- and extrachromosomal mobilome transfers resistance- and virulence-encoding genes within and between species.

To further extend the genomic characterization of the *Staphylococcaceae* data set and identify the elements promoting HGT, we performed inventory of the mobile elements in each strain, starting with the plasmids. We detected *n* = 64 circularized contigs of <300 kbp, which were attributed to 48 strains representing 8 species. These plasmids ranged in size from 3,360 (*S. simulans* IVB6174) to 55,879 bp (*S. simulans* IVB6189; Fig. S3A, Data Set S2). Out of 64 plasmids, 54 were confirmed with PlasmidFinder (25). Individual strains possessed up to three plasmids (Fig. S3A). We compared GC content between the chromosome and the plasmids to highlight GC content differences, which indicate HGT from phylogenetically unrelated species. Two S. delphini strains (out of six), IVB6222 (CP094737) and IVB6245 (CP094735), possessed plasmids with a significantly divergent GC content from the chromosomal GC content (Fig. S3A). Interestingly, we identified three PlasmidFinder-positive plasmids which were also positive in a PHASTER search (26) in the strains S. aureus IVB6165 (CP094794) and *S. simulans* IVB6192 (CP094708) and IVB6209 (CP094703) (red disks in Fig. S3A), suggesting that they are either circularized prophage genomes or plasmid-encoded prophage sequences.

Using PHASTER, we also searched systematically for the presence of prophages within the bacterial chromosomes. The genomic coordinates and scores of all genome hits are summarized in Data Set S3. All strains had at least one prophage candidate, labeled as partial (score < 70), questionable (score = 70 to 90), or complete (score > 90) according to PHASTER’s output settings (26). Prophage candidates were also found on plasmids in 12 strains, as shown in Table 2. S. aureus, *S. simulans*, S. delphini, and *S. simulans*-like strains had prophages predicted on plasmids. Most (9/12) of these plasmids were negative in a PlasmidFinder screen (Fig. S3A, Table 2). We cannot exclude the possibility that these false-negative PlasmidFinder results are due to a more distant plasmid family which is absent from the database. Functional annotations of selected plasmids encoding prophages with eggNOG-mapper (27) and RAST (28) highlighted the co-presence of plasmid features and phage signatures (replicase, lysogeny, DNA metabolism, packaging, tail, lysis), pointing to plasmids with prophages or phage-plasmid entities (29). A systematic taxonomic annotation of the predicted prophages (*n* = 370 in total) confirmed that they all belonged to the order *Caudovirales*. A more detailed family-level view showed a diverse distribution in terms of number and phage families across *Staphylococcaceae* species and strains (Fig. S3B). Most of the strains had prophage sequences of *Siphoviridae* origin (*n* = 273/370), as expected from previous studies (30). Four *Myoviridae*, which are known to sometimes have a broad host range and high potential in phage therapy (31), could also be detected in *M. sciuri* IVB6230 (*n* = 1), *S. arlettae* IVB6235 (*n* = 1), *S. ureilyticus* IVB6236 (*n* = 1), and *S. muscae-*like IVB6233 (*n* = 1) strains. Due to the complexity of phage taxonomic classification, especially when it comes to assignment of incomplete prophage sequences, a substantial number of prophages (N = 93/370) could not be unambiguously attributed to a taxonomic family and thus were labeled as “not determined.”

**TABLE 2:** Plasmid-encoded prophage sequences

Plasmid identifier	Plasmid size (kbp)	Size (kbp) of predicted phage(s)	Predicted phage genome completeness score^*a*^	Predicted phage family	PlasmidFinder
S. aureus					
IVB6154 CP095120	41	41	Intact (120)	*Siphoviridae*	–
IVB6156 CP095117	41	29	Intact (100)	*Siphoviridae*	–
IVB6157 CP095113	14	10	Incomplete (30)	*Siphoviridae*	–
IVB6165 CP094794	37	30	Incomplete (60)	Ambiguous	+
IVB6171 CP095110	39	28	Questionable (90)	*Siphoviridae*	–
S. simulans					
IVB6179 CP095105	36	26	Incomplete (40)	Ambiguous	–
IVB6188 CP095103	56	21	Questionable (70)	Ambiguous	–
IVB6189 CP095101	56	21	Questionable (70)	Ambiguous	–
IVB6192 CP094708	49	17; 10	Incomplete (50)	*Siphoviridae*	+
IVB6209 CP094703	38	18	Incomplete (40)	*Siphoviridae*	+
S. simulans-like IVB6181 CP095097	33	29	Questionable (80)	Ambiguous	–
S. delphini IVB6226 CP095108	44	43	Questionable (80)	*Siphoviridae*	–

a PHASTER scoring considers prophage as intact for a score of >90, questionable for a score of 70 to 90, and incomplete for a score of <70.

**Virulence factors encoded by plasmids and phages.** A subset of VF-encoding genes was on plasmids and linked to phage sequences, supporting the mosaic presence of VF-encoding genes, which is not always in line with the phylogenetic ancestry (Fig. 4 and Fig. 5B). For example, exotoxin-type VF-encoding genes were located in the prophage regions in a number of strains. The same strains had also *isd* factors (*isdA-G*) encoding iron uptake players linked to intact prophage. Besides this, *tst-1* was phage-associated in all four bovine S. aureus strains (IVB6191, IVB6172, IVB6201, and IVB6202) and in five camel S. aureus strains (IVB6167, IVB6185, IVB6166, IVB6169, and IVB6171). Interestingly, the gene *sec*, encoding enterotoxin C in IVB6156, belonged to a prophage region predicted as intact and located on a plasmid which was not detected by PlasmidFinder. The *scn* gene, encoding a protein that inhibits the immune response, was phage-associated in two camel S. aureus strains (IVB6154 and IVB6156). Finally, the clumping factor A encoded by the *clfA* gene was phage associated in strains IVB6154 and IVB6164. The *ebp* gene coding for the elastin-binding protein was present in all the 33 S. aureus strains, with two strains having it encoded by intact prophages (IVB6170 and IVB6239). Similarly, the gene *vwb*, encoding the secreted von Willebrand factor-binding protein, which was present in all strains, as expected, was phage-associated in the strains IVB6154 and IVB6156. Finally, bi-component pore-forming toxins were phage-associated in the strains IVB6156, IVB6195, IVB6154, and IVB6171.

With respect to PAI *v*Sa*β*, we performed a gene neighborhood analysis using FlaGs (32) and combined it with a core genome-based phylotree (Fig. 5B). While the genomic neighborhood indicated good synteny downstream of the *DUF4352* gene (double black arrow), the *v*Sa*β* PAI region itself showed diversity across the 33 S. aureus strains with respect to PAI structure and content. Interestingly, the four bovine S. aureus strains, which clustered distinctly in the phylotree, possessed enterotoxins and leukotoxins, while the PAIs in the camel strains possessed either one of the latter. Overall, the disagreement between the core genome phylotree and the PAI composition and structure the highlighted the dynamic remodeling of this region. Finally, we could also detect the presence of a type I restriction-modification system along with the virulence and resistance genes in this *v*Sa*β* PAI region in all the S. aureus strains (except the four strains with an uncharacterized prophage region: IVB6163, IVB6196, IVB6242, and IVB6243).

**FIG 6:**
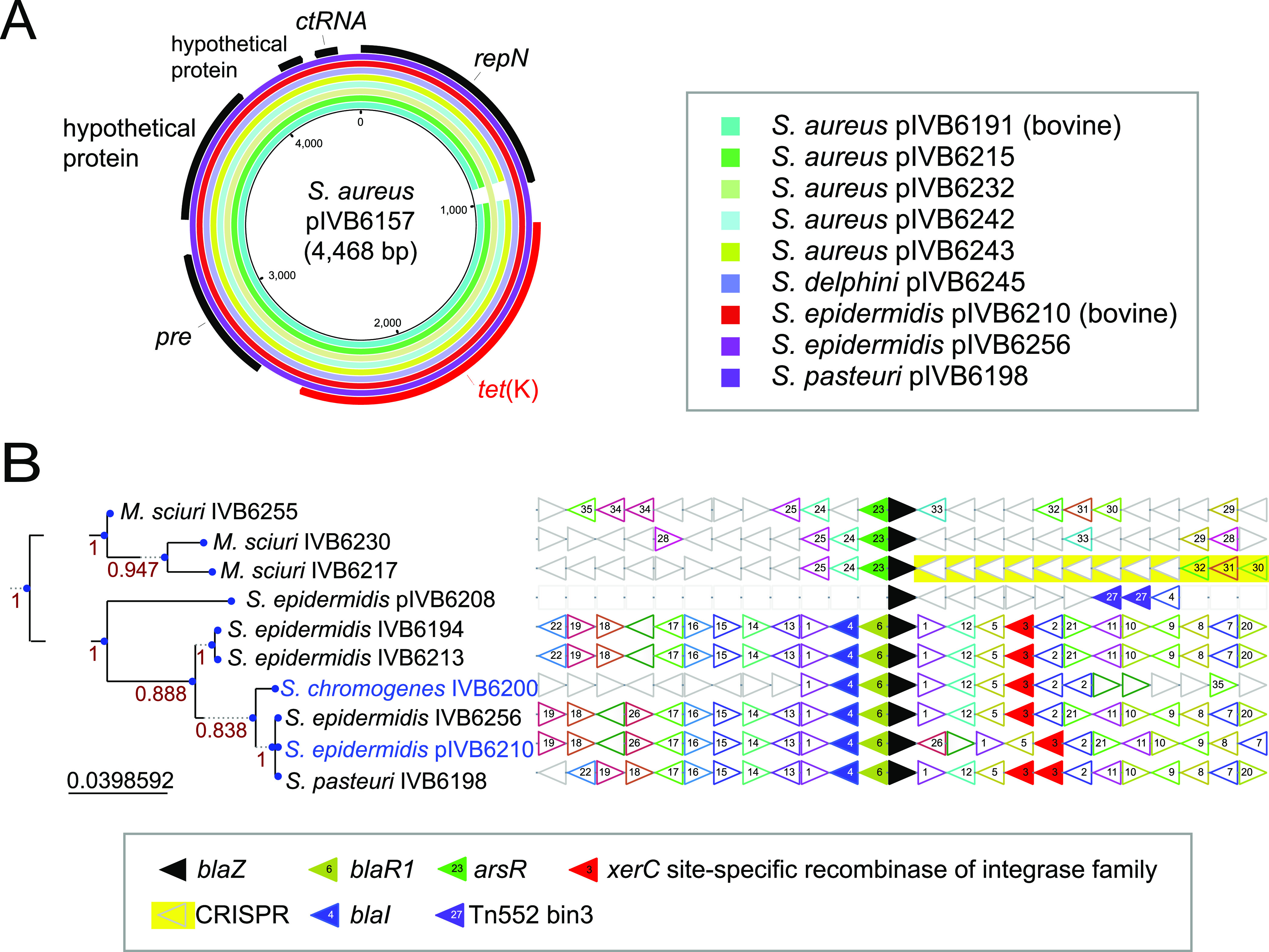
Tetracycline-encoding plasmids and signatures of horizontal gene transfer related to beta-lactam resistance. (A) Tetracycline resistance plasmids. *tet*(K)-harboring plasmids found in different *Staphylococcaceae* species, showing overall similarity and synteny. (B) Beta-lactam resistance gene *blaZ* neighborhood analysis. The pruned tree on the left is based on the translated aa sequences of *blaZ.* Horizontal gene transfer of *blaZ* is indicated since the species harboring the gene do not cluster together.

**Resistance mobilome.** All the *tet*(K) genes detected in S. aureus, S. epidermidis, S. delphini, and *S. simulans* showed high sequence conservation and were plasmid-encoded (Fig. 6A). The *tet*(K)-encoding plasmid of S. delphini had a size of 4.5 kbp and has not been reported before for this species. The strains with a *tet*(K)-encoding pT181-like plasmid were derived from samples collected in different years and regions in Kenya and from different specimens (milk from mastitis, nasal swab from tick bite, pus swab from wound; Data Set S1), including one strain sampled from cattle (S. aureus IVB6191) supporting the horizontal transfer of *tet*(K). In summary, the *tet*(K)-containing plasmid was present in different strains of different Staphylococcus species isolated from both camels and cattle, indicating its widespread occurrence in East Africa.

The *blaZ* resistance gene encoding the beta-lactamase or penicillinase protein was detected in coagulase-negative *M. sciuri*, S. epidermidis, *S. pasteuri*, and *S. chromogenes*, and its presence correlated 100% with the non-wild-type phenotype against benzylpenicillin (Fig. 2). The gene was chromosomally located in all strains except S. epidermidis IVB6208, where it was plasmid-encoded (Fig. 2 and Fig. 6B). In *S. chromogenes* IVB6200, *blaZ* localized (Data Set S1) within a prophage region (Data Set S3). A genetic context analysis of *blaZ* insertions clearly pointed toward dynamic lateral genetic exchanges (Fig. 6B) between coagulase-negative species. The *M. sciuri blaZ* and flanking genes formed a distinct group, while S. epidermidis, *S. chromogenes*, and *S. pasteuri* sequences clustered in a second main branch with a conserved synteny. While S. epidermidis and *S. pasteuri* belonged to the clade D phylogroup, *S. chromogenes* was part of the clade B species group of *Staphylococcaceae* (Fig. 1B). In line with HGT and disruptive phylogenies, the *blaZ*-based phylotree (Fig. 6B) showed *S. chromogenes* IVB6200 clustering with S. epidermidis IVB6210 and IVB6256, while S. epidermidis IVB6208, IVB6194, and IVB6213 formed a distinct subgroup. It is also notable that *S. chromogenes* IVB6200 was sampled from a cattle host. We also confirmed the presence of *bla_ARL_*, encoding a group II beta-lactamase, which was recently first described in our *S. arlettae* (33) strains. Interestingly, *mecA* in S. epidermidis IVB6208, present at the chromosome position 50,915 to 52,921 (Data Set S1), was associated with a prophage (Data Set S3). An *in silico* screen with the webtool SCC*mec*Finder did not reveal an intact SCC*mec* cassette but the *mec* classB gene. The latter was flanked upstream by *ccrB4* as well as *IS1272* and downstream by *dmecR1, ccrB4*, and *IS1272*. The nearest SCC*mec* cassette type was SCC*mec* type VI(4B).

### Toxin-antitoxin system distribution in *Staphylococcaceae*.

**FIG 7:**
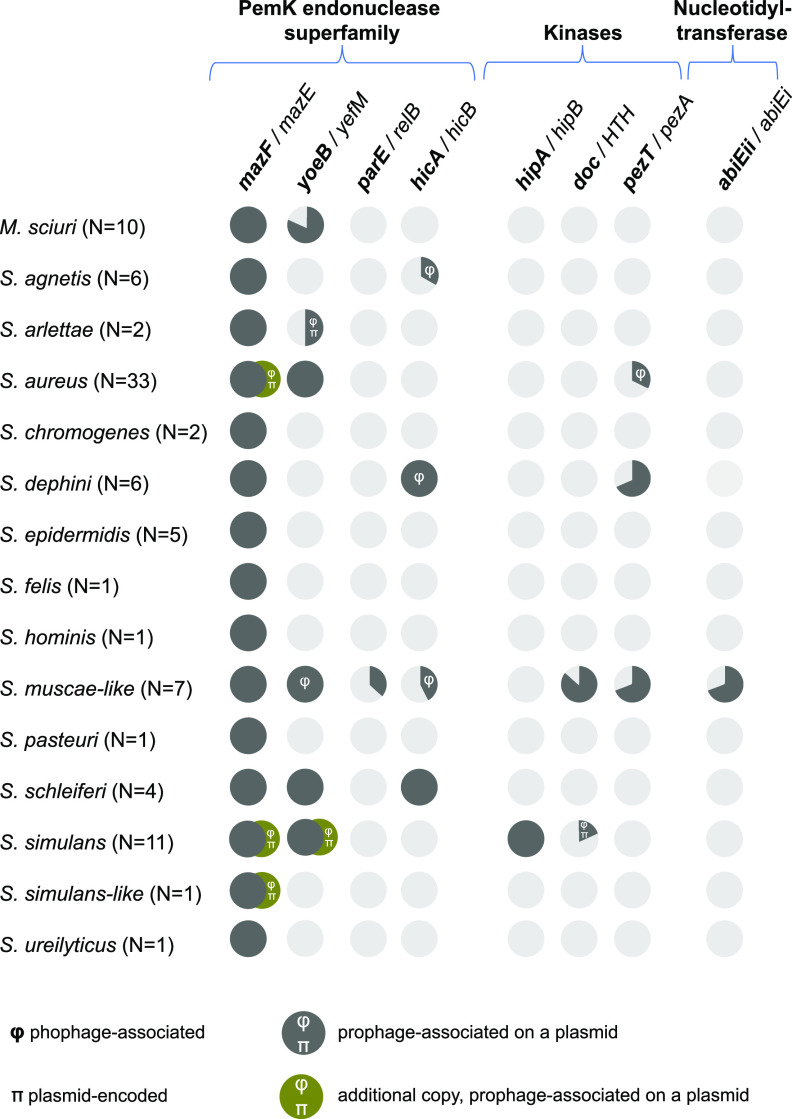
Toxin-antitoxin (TA) systems in *Staphylococcaceae*. Putative TA pairs were identified in the 91 *Staphylococcaceae* strains and classified into the corresponding families using the TASmania database. Genes in bold indicate toxin-encoding elements. The symbol π denotes plasmid-encoded TA pairs; the symbol φ indicates phage-encoded TA pairs.

TA systems are at the crossroads of the resistome and virulome and can also be transferred horizontally via plasmids and phages. Using the hidden Markov models (HMM) profiles from TASmania (34) combined with the guilt-by-association method, we mapped the putative TAs and analyzed their distribution across the whole genomes of the 91 strains (Data Set S4 and Fig. 7). The *mazF/mazE* system was systematically present in the chromosomes of all 91 genomes, as expected from the literature (34, 35). We also identified a second *mazF/mazE* TA system associated with a phage sequence on a 40-kbp plasmid in S. aureus IVB6154, IVB6156, and IVB6171 (Data Sets S3 and S4). Whether the chromosomal and plasmid-encoded copies interfere with each other is unknown, but the phage-containing plasmid is likely to mobilize the *mazF/mazE* TA system. We mapped the *yoeB/yefM* system in all S. aureus, *S. muscae*-like, *S. schleiferi*, and *S. simulans* strains, as well as in 1 *S. arlettae* strain and 8 out of 10 of *M. sciuri* strains. Our screen also identified a putative *hicA/hicB* TA system (which is peculiar for having the toxin upstream of the antitoxin) in *S. agnetis* (2 out of 7), S. delphini (6/6), *S. muscae*-like (3/7) and *S. schleiferi* (4/4) strains. To the best of our knowledge, this TA system has never been described before in these species. A rapid Pfam search with these *hicA* sequences also revealed the PF07927 (HicA_toxin family) as a significant hit. A putative *hipA/hipB* module was detected in all *S. simulans* strains in our data set, which was unexpected because this TA system has not been previously described in this species. The putative *pezT/pezA* system was found heterogeneously in S. aureus, S. delphini, and *S. muscae*-like species of our data set. Surprisingly, we found some *abiEii/abiEi* modules—TA systems of type IV—in 3/7 *S. muscae*-like strains, appearing as an operon composed of a putative antitoxin (*abiEi*) followed by two putative toxins (*abiEii*). In summary, the East African strains, which are mostly phylogenetically distinct from other strains of the same species, harbor novel TA systems.

### Methylomes of the strains.

We investigated the methylome status of the 91 genomes to trace potential signs of HGT and the regulation of expression of specific gene sets. We took advantage of the PacBio Single Molecule Real Time (SMRT) sequencing technology, which allows the tracking of ^m6^A, ^m4^C, and ^m5^C DNA modifications. We identified and quantified the presence of 121 consensus motifs around the sites of these modifications (Data Set S4). We then inferred the putative methylases (MTases; Data Set S4) and their associated restriction enzyme (RE) whenever applicable. We added strain-specific core genome-based phylotrees to the strain-specific methylation profiles to highlight the intra- and interspecies conservation and diversity of these profiles (Fig. S4A). As expected from the characteristics of SMRT sequencing (i.e., higher sensitivity for ^m6^A methylation), most of the reported motifs had ^m6^A modification, as found in long non-palindromic type I motifs (bipartite ones with stretch of Ns) or short palindromic type II motifs such as G^m6^ATC. We detected five motifs with a ^m4^C modification, e.g., motif ^m4^CCTTC. Some methylated motifs were present across different species: G^m6^ATC in S. aureus, *S. simulans*-like IVB6181, S. delphini, and *M. sciuri* strains; GA^m6^AYNNNNNNTAGA/TCT^m6^ANNNNNNRTTC in S. aureus and S. hominis strains; G^m6^ATGC/GC^m6^ATC in *S. simulans*-like IVB6181 and *S. arlettae* strains; and CCA^m6^AG in *M. sciuri* and *S. muscae*-like strains. The remaining motifs were species- or strain-specific. Most of the motifs presented high levels of methylation percentage (>99%) across the genome. Modification levels of >99% are usually associated, in combination with a restriction enzyme, with host protection against invading phages. Therefore, we investigated the potential association between prophage sequences and motif distribution by focusing on the G^m6^ATC presence identified across nine strains encompassing four species, plus the *S. simulans*-like IVB6181 strain (Fig. S4B to E). Overall, we did not find a compelling correlation between prophage and G^m6^ATC motif distribution, except for some G^m6^ATC motif depletion at predicted phage sequence regions in S. delphini IVB6190 and IVB6245 (Fig. S4F and G), plus one prophage region in *M. sciuri* IVB6223 (Fig. S4B). We analyzed the conservation degree of the candidate restriction-modification (RM) system of G^m6^ATC motif. The RM system possessed two adjacent MTases (*dpnM* and *dpnA*) on the chromosomes of S. delphini IVB6190 and IVB6245 and *S. simulans* IVB6192, and on a plasmid of S. aureus IVB6164. A single MTase was present on a plasmid of the *S. simulans*-like IVB6181 where the RM system overlapped with a prophage sequence. The candidate MTase for the G^m6^ATC motif appeared as potential orphan (i.e., a RE was not predicted in the vicinity) in the chromosome of *M. sciuri* strains (IVB6223, IVB6224, IVB6225, IVB6228, and IVB6229) (Fig. S4B). Intriguingly, although the RM system was identical (100% in amino acid sequence) between S. delphini IVB6190 and IVB6245—as expected since these two strains were phylogenetically closely related (Fig. S1D)—the corresponding G^m6^ATC motif and prophage region distribution differed in the φ2 region in IVB6190 and the φ2/φ3 region in IVB6245 (Fig. S4F and G).

The methylome, when overlapped with the phage scan and virulence factors analysis as shown in previous sections, supported HGT, as illustrated with the φ1 region in S. delphini IVB6245 (Fig. S4G). This φ1 region encodes the *hlgA/hlgB* bi-component pore-forming toxin (genomic coordinates 1,117,323 to 1,119,111) and the low number of GATC motifs in this region illustrated the recent insertion of this prophage sequence in the S. delphini IVB6245 chromosome. Similarly, the φ3 region of S. delphini strain IVB6245 contained the *sec* gene (genomic coordinates 2,104,351 to 2,105,051) and was also depleted of its GATC motifs.

## DISCUSSION

Our *Staphylococcaceae* data set of 91 genomes, encompassing 13 known species and 7 strains which could not be assigned to any known species, substantially increased the current pool and diversity of publicly available complete genomes, especially for the species S. delphini. To the best of our knowledge, this is the first report of isolation of the species *S. agnetis*, *S. arlettae*, *S. ureilyticus*, S. delphini, *S. felis*, *M. sciuri*, *S. muscae*-like, *S. pasteuri*, *S. schleiferi*, and *S. simulans* from dromedary camels. Of the isolated species in this study, *S. agnetis*, *S. arlettae*, S. aureus, *S. ureilyticus*, S. delphini, S. epidermidis, *S. felis*, S. hominis, *M. sciuri*, *S. pasteuri*, *S. schleiferi*, *S. simulans*, and *S. simulans*-like have been associated with disease. The strains investigated likely represent transient or longer-term residents of the animals’ flora, but they could also be human-acquired. An association of strains with disease does not necessarily mean that they are the causative agent of disease.

Many strains of this study had STs which are unique to camels, which is in line with the ecological niche dromedary camels occupy and the limited scientific data available for them. However, due to the paucity of data from pastoralists and their livestock species, it is likely that the camel STs also occur in other species. Four S. aureus strains were of ST-2957, which belongs to the CC30 and is shared with human strains. The same applies to S. epidermidis strains with the ST profiles ST-6 and ST-251 which are in common with human strains, indicating a potential risk to human health in this resource-poor region of the world. However, shared STs across hosts does not necessarily indicate zoonotic potential, e.g., as shown for Streptococcus agalactiae (8, 36). Clarification of zoonotic risks of camel staphylococci would require comparison with human strains from diseased people in the same region, which is problematic due to the minimal or absent medical infrastructure in resource-poor settings. Even though the core genome-based phylogenetic trees with camel strains scattered across the branches (Fig. S1A to F) did not indicate a strict camel-specific lineage for a given *Staphylococcaceae* species, we often observed camel strains clustering distinctively in a monophyletic manner. For example, four out of six camel strains clustered together in *S. agnetis* (Fig. S1B), and several camel clusters in S. aureus formed monophyletic-like subgroups (Fig. S1C), as did camel strains from S. delphini (Fig. S1D) and *S. simulans* (Fig. S1F). The *S. agnetis* phylotree (Fig. S1B) confirmed previous findings that the poultry-derived strains all clustered in a separate clade along with some cattle strains (19).

The camels investigated in this study belonged to pastoralists and roamed freely, and therefore represented extensive camel keeping in contrast to the camel keeping in most countries of the Arabian Peninsula. First, we wanted to compare levels of antimicrobial resistance or, more precisely, levels of wild-type and non-wild-type populations, using phenotypic and genotypic testing. Our study reports the first multidrug-resistant S. epidermidis strain of ST-1136 isolated from a camel, showing a non-wild-type phenotype toward three classes of antimicrobials: namely, tetracycline, trimethoprim, and benzylpenicillin (Fig. 2). Kenyan strains showed resistance to more antimicrobials, which is likely the result of better access to antimicrobials in remote regions of Kenya compared to the less economically developed regions of Somalia/Somaliland.

Ten out of the fourteen known species—corresponding to *n* = 34 strains, or 37% of the data set—had at least one strain with a resistance gene. More specifically, 10 strains (10.9% of this data set) harbored a *blaZ* resistance gene on the chromosome (9/10) or a plasmid (1/10), concordant with the strain’s AST phenotyping (Fig. 2). A previous work performed on 45 camel S. aureus strains from Dubai, where camel farming is rather intensive, showed that 6.67% were resistant and beta-lactamase operon-positive (10). Also, 16 strains in this study presented a plasmid-encoded *tet*(K) gene. The overall *tet*(K) prevalence of 17% in this study (27% for S. aureus) is higher than that reported for camel S. aureus in Dubai. Such levels of *tet*(K) presence in strains isolated from camels kept extensively are alarming, since tetracycline is one of the main affordable antimicrobials for the resource-poor, livestock-dependent people in sub-Saharan Africa. A study of camel S. agalactiae strains from East Africa (8) showed that 34% harbored a functional *tet*(M) gene on a Tn*916*-like transposon in comparison. ARG spillover, even between more distantly related bacterial species, is likely to be fostered—even if total antimicrobial use in the Horn of Africa is low compared to that in developed countries—by inappropriate use of antimicrobials, counterfeit-type antimicrobials, and inappropriately stored drugs, three scenarios that have been reported in many locations in sub-Saharan Africa. As expected, we detected compelling more virulence-encoding genes in the opportunistic pathogen S. aureus compared to the other coagulase-negative species we investigated.

Our mobilome analysis of East African *Staphylococcaceae* strains showed that the ARG pool (Fig. 2), TA systems (Fig. 7 and Data Set S4), and virulence factors, such as exotoxins in particular (Fig. 4), were often associated with plasmid and phage sequences, the latter being present at both chromosomal and extrachromosomal levels. We identified signatures of horizontal ARG transfer between camel and cattle strains, in particular for *tet*(K) (Fig. 6A) and *blaZ* (Fig. 6B). Each genome analyzed contained at least one integrated phage genome; ARGs and VF-encoding genes related to the pathogenicity island *v*Sa*β* in the S. aureus genomes showed how phages shuffled virulence and resistance factor-encoding genes (Fig. 5B).

The TA systems were the third family of genes we investigated via *in silico* screens (Fig. 7), which identified TA candidates never described in some of the *Staphylococcaceae* species and which will be worth further experimental testing. *S. muscae*-like strains in particular harbored a strikingly broad range of TA system families, which has never been mentioned before in the literature, showing the importance of the genomes in this study for public database diversity.

Finally, we extracted DNA methylation information for the ^m6^A and ^m^4C nucleotides and inferred the level of genome-wide methylation for the motifs where these modifications could be found. We detected different types of motifs for each genome and observed strain-specific trends for most of these methylated motifs (Fig. S4A). Nevertheless, certain methylated motifs were common to different strains and even different species, as highlighted by the type II short palindromic G^m6^ATC methylation. Intriguingly, only IVB6164 in the S. aureus subset showed G^m6^ATC methylation, raising questions about the roles and regulations of these loci and sequences in the closest relatives of IVB6164 such as IVB6232 (Fig. S1C). Future comparative studies of gene expression and regulation might use the data provided here to obtain more insights into the effects of methylation on non-coding regions such as promoters. In particular, the motifs with lower methylation ratios, e.g., G^m6^AARVNSNGTC in the two S. delphini strains IVB6219 and IVB6226 (Fig. S4A), might be a good starting point for such analysis. We had performed an initial round of MTase screening (Data Set S4) potentially associated with the observed methylation profiles, and a pathogenicity island analysis in S. aureus (Fig. 5B) highlighted the presence of a type I restriction modification system within the mobile regions of phage origin which also included transposases. A follow-up of this would be the comparative study of the conservation/diversity of putative MTases across these strains.

Altogether, our study addressed strains from East African dromedary camels, an ecological niche which is less investigated than other livestock species. We detected several known *Staphylococcaceae* species and several strains representing novel species, which will be investigated in future studies. Many strains of the species formed specific clusters. The different coagulase-negative species investigated contained subsets of resistance genes and VF-encoding genes and are a reservoir for the latter to other bacteria. Further work is needed to shed more light on the nature and roles of different *Staphylococcaceae* species in dromedary camels.

## MATERIALS and METHODS

### Isolation of *Staphylococcaceae* strains used in this study and species-level identification.

Information about the different strains used in this study is provided in Data Set S1 in the supplemental material. Samples were either collected in the framework of routine diagnostic procedures or as part of investigations on a permit issued by the accredited Institutional Animal Care and Use Committee of the International Livestock Research Institute in Nairobi, Kenya (approval no. IACUC 2014.8). The *Staphylococcaceae* strains were isolated using standard methods (37) without any enrichment to select for high MICs and stored for subsequent use at −80°C. Species designation of the various strains was first performed via MALDI-TOF MS analysis and then, once the draft genomes were obtained (see below), confirmed by genomic sequence analysis using the type strain genome server (TYGS) (18). For MALDI-TOF MS analysis, strains were streaked onto trypticase soy agar with 5% sheep blood (TSA-B; Becton, Dickinson and Co.) and incubated at 37°C overnight. A small amount of colony material was transferred onto a steel target plate, 1 μL of 70% formic acid was added to the colony material, and it was allowed to air dry before 1 μL of HCCA matrix solution (α-cyano-4-hydroxycinnamic acid; Bruker Daltonics) was added. MALDI-TOF MS measurements to identify the species were performed using a Microflex LT instrument (Bruker Daltonics) and the MBT 7854 MSP Library.

### Phenotypic characterization and antimicrobial susceptibility testing.

Strains were grown on TSA-B at 37°C overnight. Samples to be run on the VITEK 2 GP identification card (bioMérieux) via the automated VITEK 2 Compact system (bioMérieux) were prepared according to manufacturer’s recommendations. Likewise, the antimicrobial susceptibility tests (AST) were performed using the Gram-positive susceptibility card VITEK 2 AST-GP80 (bioMérieux) on the VITEK 2 Compact system according to manufacturer’s recommendations. Strains which showed non-wild-type MICs in the VITEK 2 AST-GP80 were subsequently subjected to MIC determination by broth microdilution in cation-adjusted Mueller-Hinton broth (CAMHB) using Sensititre custom-made plates (Thermo Fisher Scientific). The plates were incubated under aerobic conditions at 35 ± 1°C for 16 to 20 h. MIC values were interpreted using epidemiological cutoff values (ECOFFs) published by the European Committee on Antimicrobial Susceptibility Testing (EUCAST) for S. aureus or coagulase-negative staphylococci (MIC distribution and ECOFF database, accessed 11 July 2022; www.eucast.org) except for oxacillin and trimethoprim-sulfamethoxazole in coagulase-negative staphylococci, for which we followed CLSI recommendations (CLSI supplement M100; CLSI, 2021). The MICs of the following antimicrobials were tested for all strains: penicillin, ampicillin, amoxicillin-clavulanic acid (ratio 2:1), oxacillin, cefoxitin, cefalothin, cefovecin, cefpodoxime, ceftiofur, cefoperazone, cefotaxime, cefquinome, gentamicin, ciprofloxacin, marbofloxacin, enrofloxacin, trimethoprim/sulfamethoxaozole (ratio 1:20), tetracycline, erythromycin, clindamycin, pirlimycin, linezolid, vancomycin, quinupristin-dalfopristin, and rifampicin. Trimethoprim and streptomycin were tested for one S. epidermidis and one *S. simulans* strain, respectively. Non-wild-type phenotypes indicated resistance.

### Isolation of high-molecular weight genomic DNA.

Genomic DNA was basically isolated as described elsewhere (38). Briefly, the strains were grown on TSA-B at 37°C overnight. A loop of colony material was suspended in 500 μL of 0.85% NaCl solution and centrifuged at 21,300 × *g* for 5 min. The supernatant was discarded and the cell pellet resuspended in 100 μL of suspension buffer (20 mM Tris [pH 8.0], 2 mM EDTA, 1.2% Triton X-100, 20 mg/mL lysozyme); 2 μL of lysostaphin (5 mg/mL) was added and the solution was incubated at 37°C for 30 min. Cells were further lysed by adding 500 μL of GES solution (5 M guanidium thiocyanate, 0.5 M EDTA, 0.5% sarcosyl) and 20 μL of proteinase K (20 mg/mL) followed by incubation at 60°C for 30 min. The lysate was cooled on ice before 250 μL of ice-cold 7.5 M ammonium acetate was added. The lysate was mixed thoroughly and incubated on ice for 10 min. Afterwards, 500 μL of phenol:chloroform:isoamyl alcohol (25:24:1) mixture was added, the phases were mixed thoroughly, and the tube was centrifuged at 21,300 × *g* at 4°C for 15 min. The supernatant was transferred in a new tube and 0.54 vol of ice-cold isopropanol was added and mixed. Genomic DNA precipitated and was centrifuged for 1 min at 6,500 × *g*. The pellet was washed two times in 70% ethanol and dried at room temperature (RT) for 5 min. The DNA sample was dissolved in 150 μL TE buffer (Tris 10 Mm [pH 8.0], 1 mM EDTA), then 1 μL of RNase A (100 mg/mL) was added and the mixture was incubated at RT for 30 min. Finally, the DNA solution was purified using the DNeasy PowerClean Cleanup kit (Qiagen), eluted in a final volume of 100 μL TE (pH 8.0), and stored at −80°C for subsequent use. The genomic DNA quality and quantity was measured by a QuDye dsDNA HS Assay kit (Invitrogen) on a Qubit 3 Fluorometer (Invitrogen).

### Whole-genome sequencing.

Library preparations and sequencing were performed at Lausanne Genomic essentially as recently described (39). Briefly, high-molecular weight DNA was sheared with Megaruptor (Diagenode, Denville, NJ, USA) to obtain 10- to 15-kb fragments. After shearing the DNA, size distribution was checked on a Fragment Analyzer (Advanced Analytical Technologies, Ames, IA, USA). A total of 500 ng of DNA was used to prepare a SMRTbell library with the PacBio SMRTbell Express Template Prep kit 2.0 (Pacific Biosciences, Menlo Park, CA, USA) according to the manufacturer’s recommendations. The resulting library was pooled with other libraries which were processed in the same manner. The pool was size-selected with Ampure PacBio beads to eliminate fragments of <3kb. It was sequenced with v2.0/v2.0 chemistry and diffusion loading on a PacBio Sequel II instrument (Pacific Biosciences, Menlo Park, CA, USA) at a 900-min movie length and a pre-extension time of 120 min using one SMRT cell 8 M.

### Bioinformatic analysis.

All bioinformatics analysis was performed on local Ubuntu machines (18.04 LTS and 20.04 LTS) and servers of the IBU Linux Cluster from Bern, using custom bash, R, and Perl scripts. Assemblies and annotations were basically done as recently reported (39). Default parameters were used for all software applications unless stated otherwise. Assemblies were done using Flye software (v2.6 release) (15). Contigs were circularized and used for downstream analysis. The draft assemblies were polished with three rounds of the software arrow (SMRTLink8 package). The chromosomes were rotated using the gene *dnaA*. Annotations, tRNA predictions, and coding sequences (CDS) predictions were done using Prokka v1.13 (40), ARAGORN (41), and RNAmmer software (42) and Prodigal (43), respectively. PHASTER (26) was used to scan chromosomes in order to locate prophage sequences. The non-chromosomal circularized contigs were selected and screened for potential plasmids with PlasmidFinder (v2.1.1) (25) and for phage identification with PHASTER. Prokka, eggNOG-mapper (v2.1.6) (27), and RAST (28) annotation tools were used to further investigate the functional signatures of these putative plasmid and phage molecules. Average nucleotide identity (ANI) was computed with FastANI software (v1.3, default settings) (16) in all-against-all pairwise comparison of the 91 complete genomes. BRIG (24) was used to draw whole-genome comparisons ring plots. The webtool SCC*mec*Finder (https://cge.food.dtu.dk/services/SCCmecFinder/) was used to screen for *mecA*-associated SCC*mec* cassettes (44).

### 16S rRNA gene and core genome-based phylogenetics analysis.

The 16S rRNA gene-based phylotree was built from the full-length gene sequence alignment with MUSCLE (3.8.1551) (45) and the PhyML program (v3.3.20180214) (46) run with 1,000 bootstraps under the GTR substitution model (47). The pan and core analyses were performed with Roary (v3.11) (48) with the main parameters set as “-i 90 -cd 90 -e –mafft -n -iv 1.5”. This output core genome alignment was fed into IQ-TREE2 (v2.0.3) (49) with parameters such as the PhyML model (-mset phyml), standard model selection (-m TEST), and its ultrafast bootstrap mode (-bb 1000 -bnni) combined with the corresponding likelihood branch test (-alrt 1000). The final maximum likelihood tree was drawn with FigTree (v1.4.4).

### Generation of minimum spanning trees using MLST data.

MLST was performed with the R package MLSTar (50) for the *Staphylococcaceae* species from our data set that have schemes available on the PubMLST database, namely, S. aureus, *S. chromogenes*, S. epidermidis, S. hominis, and *M. sciuri*. The minimum spanning trees were inferred using PHYLOViZ (v2.0) (51) with its goeBURST algorithm with default parameters (52).

### Detection of genes encoding virulence factors and antimicrobial resistance.

Virulence factors were identified with ABRicate (https://github.com/tseemann/abricate) run against its included virulence factor database (VFDB) (53). *In silico* screens for antimicrobial resistance genes were performed with ResFinder v4.0 (20). Gene neighborhood analysis was performed with FlaGs (v1.2.6) (32).

### Detection of candidate toxin-antitoxin systems.

The TA systems were identified by scanning the Prokka-predicted proteomes with the toxin and antitoxin HMM profiles downloaded from TASmania (34), using the HMMER3 command “hmmsearch -E 0.001”. The candidate toxin and antitoxin loci were then manually curated to filter off singletons and keep the TA pairs only.

### Methylation profiling.

Modified bases (^m5^C, ^m4^C, and ^m6^A) and their associated DNA motifs were identified using pb_basemods cromwell workflow and motifMaker program (SMRTLink9 package) as recommended by the manufacturer. The list of motifs was concatenated to remove duplicates and manually cleaned to remove degenerated motifs. Using a Perl script and EMBOSS fuzznuc (54), the cleaned list of motifs was reevaluated against all genomes, and the validated motifs (mean score ≥ 30 and methylated ratio > 0.2) for each genome were retained. The percentage of methylations per motif per genome was compiled by parsing the curated motifs list. The presence of methylases and restriction enzyme genes was evaluated for each genome using custom scripts. First, the Prokka annotated proteins of each genome were assessed for the presence of specific InterPro (55) domains via hmmscan (56) and EMBOSS fuzzpro (for PROSITE patterns) (54). Next, the list of hits was parsed and the motif assigned to potential methylase.

### Ethics approval and consent to participate.

All protocols of this study were designed and performed in strict accordance with Kenyan legislation for animal experimentation and were approved by the Institutional Animal Care and Use Committee of the International Livestock Research Institute (IACUC reference no. 2014.08 and 2015.08).

### Data availability.

The sequences and annotations of the chromosomes and plasmids are available in the NCBI under project no. PRJNA819273.

## References

[1] Faye B. 2014. The camel today: assets and potentials. Anthropozoologica 49:167–176. 10.5252/az2014n2a01.

[2] Faye B, Chaibou M, Vias G. 2012. Integrated impact of climate change and socioeconomic development on the evolution of camel farming systems. Bjecc 2:227–244. 10.9734/BJECC/2012/1548.

[3] Zarrin M, Riveros JL, Ahmadpour A, de Almeida AM, Konuspayeva G, Vargas-Bello-Perez E, Faye B, Hernandez-Castellano LE. 2020. Camelids: new players in the international animal production context. Trop Anim Health Prod 52:903–913. 10.1007/s11250-019-02197-2. 31898022

[4] Younan M, Abdurahman O. 2004. Milk hygiene and udder health, p 67–76. *In* Farah Z, Fischer A (ed), Milk and meat from the camel. Hochschulverlag AG an der ETH, Zurich, Switzerland.

[5] Corman VM, Jores J, Meyer B, Younan M, Liljander A, Said MY, Gluecks I, Lattwein E, Bosch BJ, Drexler JF, Bornstein S, Drosten C, Muller MA. 2014. Antibodies against MERS coronavirus in dromedary camels, Kenya, 1992–2013. Emerg Infect Dis 20:1319–1322. 10.3201/eid2008.140596. 25075637 PMC4111164

[6] Muller MA, Corman VM, Jores J, Meyer B, Younan M, Liljander A, Bosch BJ, Lattwein E, Hilali M, Musa BE, Bornstein S, Drosten C. 2014. MERS coronavirus neutralizing antibodies in camels, Eastern Africa, 1983–1997. Emerg Infect Dis 20:2093–2095. 10.3201/eid2012.141026. 25425139 PMC4257824

[7] Rasche A, Saqib M, Liljander AM, Bornstein S, Zohaib A, Renneker S, Steinhagen K, Wernery R, Younan M, Gluecks I, Hilali M, Musa BE, Jores J, Wernery U, Drexler JF, Drosten C, Corman VM. 2016. Hepatitis E virus infection in dromedaries, North and East Africa, United Arab Emirates, and Pakistan, 1983–2015. Emerg Infect Dis 22:1249–1252. 10.3201/eid2207.160168. 27315454 PMC4918144

[8] Fischer A, Liljander A, Kaspar H, Muriuki C, Fuxelius HH, Bongcam-Rudloff E, de Villiers EP, Huber CA, Frey J, Daubenberger C, Bishop R, Younan M, Jores J. 2013. Camel *Streptococcus agalactiae* populations are associated with specific disease complexes and acquired the tetracycline resistance gene *tetM* via a Tn*916*-like element. Vet Res 44:86. 10.1186/1297-9716-44-86. 24083845 PMC3850529

[9] Jans C, Merz A, Johler S, Younan M, Tanner SA, Kaindi DWM, Wangoh J, Bonfoh B, Meile L, Tasara T. 2017. East and West African milk products are reservoirs for human and livestock-associated *Staphylococcus aureus*. Food Microbiol 65:64–73. 10.1016/j.fm.2017.01.017. 28400021

[10] Monecke S, Ehricht R, Slickers P, Wernery R, Johnson B, Jose S, Wernery U. 2011. Microarray-based genotyping of *Staphylococcus aureus* isolates from camels. Vet Microbiol 150:309–314. 10.1016/j.vetmic.2011.02.001. 21353401

[11] Madhaiyan M, Wirth JS, Saravanan VS. 2020. Phylogenomic analyses of the *Staphylococcaceae* family suggest the reclassification of five species within the genus *Staphylococcus* as heterotypic synonyms, the promotion of five subspecies to novel species, the taxonomic reassignment of five *Staphylococcus* species to *Mammaliicoccus* gen. nov., and the formal assignment of *Nosocomiicoccus* to the family *Staphylococcaceae*. Int J Syst Evol Microbiol 70:5926–5936. 10.1099/ijsem.0.004498. 33052802

[12] Gonzalez-Martin M, Corbera JA, Suarez-Bonnet A, Tejedor-Junco MT. 2020. Virulence factors in coagulase-positive staphylococci of veterinary interest other than *Staphylococcus aureus*. Vet Q 40:118–131. 10.1080/01652176.2020.1748253. 32223696 PMC7178840

[13] Schoenfelder SMK, Dong Y, Fessler AT, Schwarz S, Schoen C, Köck R, Ziebuhr W. 2017. Antibiotic resistance profiles of coagulase-negative staphylococci in livestock environments. Vet Microbiol 200:79–87. 10.1016/j.vetmic.2016.04.019. 27185355

[14] Silva V, Canica M, Manageiro V, Verbisck N, Tejedor-Junco MT, Gonzalez-Martin M, Corbera JA, Poeta P, Igrejas G. 2022. *Staphylococcus aureus* and methicillin-resistant coagulase-negative staphylococci in nostrils and buccal mucosa of healthy camels used for recreational purposes. Animals 12:1255. 10.3390/ani12101255. 35625101 PMC9138023

[15] Kolmogorov M, Yuan J, Lin Y, Pevzner PA. 2019. Assembly of long, error-prone reads using repeat graphs. Nat Biotechnol 37:540–546. 10.1038/s41587-019-0072-8. 30936562

[16] Jain C, Rodriguez-R LM, Phillippy AM, Konstantinidis KT, Aluru S. 2018. High throughput ANI analysis of 90K prokaryotic genomes reveals clear species boundaries. Nat Commun 9:5114. 10.1038/s41467-018-07641-9. 30504855 PMC6269478

[17] Chun J, Oren A, Ventosa A, Christensen H, Arahal DR, da Costa MS, Rooney AP, Yi H, Xu XW, De Meyer S, Trujillo ME. 2018. Proposed minimal standards for the use of genome data for the taxonomy of prokaryotes. Int J Syst Evol Microbiol 68:461–466. 10.1099/ijsem.0.002516. 29292687

[18] Meier-Kolthoff JP, Goker M. 2019. TYGS is an automated high-throughput platform for state-of-the-art genome-based taxonomy. Nat Commun 10:2182. 10.1038/s41467-019-10210-3. 31097708 PMC6522516

[19] Shwani A, Adkins PRF, Ekesi NS, Alrubaye A, Calcutt MJ, Middleton JR, Rhoads DD. 2020. Whole-genome comparisons of *Staphylococcus agnetis* isolates from cattle and chickens. Appl Environ Microbiol 86:e00484-20. 10.1128/AEM.00484-20. 32245765 PMC7267197

[20] Florensa AF, Kaas RS, Clausen PLTC, Aytan-Aktug D, Aarestrup FM. 2022. ResFinder: an open online resource for identification of antimicrobial resistance genes in next-generation sequencing data and prediction of phenotypes from genotypes. Microb Genom 8:000748. 10.1099/mgen.0.000748. PMC8914360 35072601

[21] Spaan AN, van Strijp JAG, Torres VJ. 2017. Leukocidins: staphylococcal bi-component pore-forming toxins find their receptors. Nat Rev Microbiol 15:435–447. 10.1038/nrmicro.2017.27. 28420883 PMC5621924

[22] Tam K, Torres VJ. 2019. *Staphylococcus aureus* secreted toxins and extracellular enzymes. Microbiol Spectr 7:10.1128/microbiolspec.GPP3-0039-2018. 10.1128/microbiolspec.GPP3-0039-2018. PMC6422052 30873936

[23] Yoon SH, Park YK, Kim JF. 2015. PAIDB v2.0: exploration and analysis of pathogenicity and resistance islands. Nucleic Acids Res 43:D624–30. 10.1093/nar/gku985. 25336619 PMC4384037

[24] Alikhan NF, Petty NK, Ben Zakour NL, Beatson SA. 2011. BLAST Ring Image Generator (BRIG): simple prokaryote genome comparisons. BMC Genomics 12:402. 10.1186/1471-2164-12-402. 21824423 PMC3163573

[25] Carattoli A, Hasman H. 2020. PlasmidFinder and *in silico* pMLST: identification and typing of plasmid replicons in whole-genome sequencing (WGS). Methods Mol Biol 2075:285–294. 10.1007/978-1-4939-9877-7_20. 31584170

[26] Arndt D, Marcu A, Liang Y, Wishart DS. 2019. PHAST, PHASTER and PHASTEST: tools for finding prophage in bacterial genomes. Brief Bioinform 20:1560–1567. 10.1093/bib/bbx121. 29028989 PMC6781593

[27] Cantalapiedra CP, Hernandez-Plaza A, Letunic I, Bork P, Huerta-Cepas J. 2021. eggNOG-mapper v2: functional annotation, orthology assignments, and domain prediction at the metagenomic scale. Mol Biol Evol 38:5825–5829. 10.1093/molbev/msab293. 34597405 PMC8662613

[28] Overbeek R, Olson R, Pusch GD, Olsen GJ, Davis JJ, Disz T, Edwards RA, Gerdes S, Parrello B, Shukla M, Vonstein V, Wattam AR, Xia F, Stevens R. 2014. The SEED and the Rapid Annotation of microbial genomes using Subsystems Technology (RAST). Nucleic Acids Res 42:D206–D214. 10.1093/nar/gkt1226. 24293654 PMC3965101

[29] Pfeifer E, de Sousa JAM, Touchon M, Rocha EPC. 2021. Bacteria have numerous distinctive groups of phage-plasmids with conserved phage and variable plasmid gene repertoires. Nucleic Acids Res 49:2655–2673. 10.1093/nar/gkab064. 33590101 PMC7969092

[30] Hatoum-Aslan A. 2021. The phages of staphylococci: critical catalysts in health and disease. Trends Microbiol 29:1117–1129. 10.1016/j.tim.2021.04.008. 34030968 PMC8578144

[31] Cui Z, Guo X, Dong K, Zhang Y, Li Q, Zhu Y, Zeng L, Tang R, Li L. 2017. Safety assessment of *Staphylococcus* phages of the family *Myoviridae* based on complete genome sequences. Sci Rep 7:41259. 10.1038/srep41259. 28117392 PMC5259776

[32] Saha CK, Sanches Pires R, Brolin H, Delannoy M, Atkinson GC. 2021. FlaGs and webFlaGs: discovering novel biology through the analysis of gene neighbourhood conservation. Bioinformatics 37:1312–1314. 10.1093/bioinformatics/btaa788. 32956448 PMC8189683

[33] Andreis SN, Perreten V, Schwendener S. 2017. Novel beta-Lactamase *bla*_ARL_ in *Staphylococcus arlettae*. mSphere 2:e00117-17. 10.1128/mSphere.00117-17. 28497118 PMC5415633

[34] Akarsu H, Bordes P, Mansour M, Bigot DJ, Genevaux P, Falquet L. 2019. TASmania: a bacterial Toxin-Antitoxin Systems database. PLoS Comput Biol 15:e1006946. 10.1371/journal.pcbi.1006946. 31022176 PMC6504116

[35] Bukowski M, Lyzen R, Helbin WM, Bonar E, Szalewska-Palasz A, Wegrzyn G, Dubin G, Dubin A, Wladyka B. 2013. A regulatory role for *Staphylococcus aureus* toxin-antitoxin system PemIKSa. Nat Commun 4:2012. 10.1038/ncomms3012. 23774061

[36] Sorensen UB, Poulsen K, Ghezzo C, Margarit I, Kilian M. 2010. Emergence and global dissemination of host-specific *Streptococcus agalactiae* clones. mBio 1:e00178-10. 10.1128/mBio.00178-10. 20824105 PMC2932510

[37] Carter GR, Cole JR, Jr. 1990. Diagnostic procedures in veterinary bacteriology and mycology. Elsevier, Amsterdam, The Netherlands.

[38] Pitcher DG, Saunders NA, Owen RJ. 1989. Rapid extraction of bacterial genomic DNA with guanidium thiocyanate. Lett Appl Microbiol 8:151–156. 10.1111/j.1472-765X.1989.tb00262.x.

[39] Hill V, Akarsu H, Barbarroja RS, Cippa VL, Kuhnert P, Heller M, Falquet L, Heller M, Stoffel MH, Labroussaa F, Jores J. 2021. Minimalistic mycoplasmas harbor different functional toxin-antitoxin systems. PLoS Genet 17:e1009365. 10.1371/journal.pgen.1009365. 34673769 PMC8562856

[40] Seemann T. 2014. Prokka: rapid prokaryotic genome annotation. Bioinformatics 30:2068–2069. 10.1093/bioinformatics/btu153. 24642063

[41] Laslett D, Canback B. 2004. ARAGORN, a program to detect tRNA genes and tmRNA genes in nucleotide sequences. Nucleic Acids Res 32:11–16. 10.1093/nar/gkh152. 14704338 PMC373265

[42] Lagesen K, Hallin P, Rodland EA, Staerfeldt HH, Rognes T, Ussery DW. 2007. RNAmmer: consistent and rapid annotation of ribosomal RNA genes. Nucleic Acids Res 35:3100–3108. 10.1093/nar/gkm160. 17452365 PMC1888812

[43] Hyatt D, Chen GL, Locascio PF, Land ML, Larimer FW, Hauser LJ. 2010. Prodigal: prokaryotic gene recognition and translation initiation site identification. BMC Bioinformatics 11:119. 10.1186/1471-2105-11-119. 20211023 PMC2848648

[44] Kaya H, Hasman H, Larsen J, Stegger M, Johannesen TB, Allesoe RL, Lemvigh CK, Aarestrup FM, Lund O, Larsen AR. 2018. SCC*mec*Finder, a web-based tool for typing of staphylococcal cassette chromosome *mec* in *Staphylococcus aureus* using whole-genome sequence data. mSphere 3:e00612-17. 10.1128/mSphere.00612-17. 29468193 PMC5812897

[45] Edgar RC. 2004. MUSCLE: multiple sequence alignment with high accuracy and high throughput. Nucleic Acids Res 32:1792–1797. 10.1093/nar/gkh340. 15034147 PMC390337

[46] Guindon S, Delsuc F, Dufayard JF, Gascuel O. 2009. Estimating maximum likelihood phylogenies with PhyML. Methods Mol Biol 537:113–137. 10.1007/978-1-59745-251-9_6. 19378142

[47] Garcia-Mazcorro JF. 2013. Testing evolutionary models to explain the process of nucleotide substitution in gut bacterial 16S rRNA gene sequences. FEMS Microbiol Lett 346:97–104. 10.1111/1574-6968.12207. 23808388

[48] Page AJ, Cummins CA, Hunt M, Wong VK, Reuter S, Holden MT, Fookes M, Falush D, Keane JA, Parkhill J. 2015. Roary: rapid large-scale prokaryote pan genome analysis. Bioinformatics 31:3691–3693. 10.1093/bioinformatics/btv421. 26198102 PMC4817141

[49] Minh BQ, Schmidt HA, Chernomor O, Schrempf D, Woodhams MD, von Haeseler A, Lanfear R. 2020. IQ-TREE 2: new models and efficient methods for phylogenetic inference in the genomic era. Mol Biol Evol 37:1530–1534. 10.1093/molbev/msaa015. 32011700 PMC7182206

[50] Ferres I, Iraola G. 2018. MLSTar: automatic multilocus sequence typing of bacterial genomes in R. PeerJ 6:e5098. 10.7717/peerj.5098. 29922519 PMC6005169

[51] Nascimento M, Sousa A, Ramirez M, Francisco AP, Carrico JA, Vaz C. 2017. PHYLOViZ 2.0: providing scalable data integration and visualization for multiple phylogenetic inference methods. Bioinformatics 33:128–129. 10.1093/bioinformatics/btw582. 27605102

[52] Francisco AP, Bugalho M, Ramirez M, Carrico JA. 2009. Global optimal eBURST analysis of multilocus typing data using a graphic matroid approach. BMC Bioinformatics 10:152. 10.1186/1471-2105-10-152. 19450271 PMC2705362

[53] Chen L, Yang J, Yu J, Yao Z, Sun L, Shen Y, Jin Q. 2005. VFDB: a reference database for bacterial virulence factors. Nucleic Acids Res 33:D325–D328. 10.1093/nar/gki008. 15608208 PMC539962

[54] Rice P, Longden I, Bleasby A. 2000. EMBOSS: the European Molecular Biology Open Software Suite. Trends Genet 16:276–277. 10.1016/s0168-9525(00)02024-2. 10827456

[55] Mitchell AL, Attwood TK, Babbitt PC, Blum M, Bork P, Bridge A, Brown SD, Chang HY, El-Gebali S, Fraser MI, Gough J, Haft DR, Huang H, Letunic I, Lopez R, Luciani A, Madeira F, Marchler-Bauer A, Mi H, Natale DA, Necci M, Nuka G, Orengo C, Pandurangan AP, Paysan-Lafosse T, Pesseat S, Potter SC, Qureshi MA, Rawlings ND, Redaschi N, Richardson LJ, Rivoire C, Salazar GA, Sangrador-Vegas A, Sigrist CJA, Sillitoe I, Sutton GG, Thanki N, Thomas PD, Tosatto SCE, Yong SY, Finn RD. 2019. InterPro in 2019: improving coverage, classification and access to protein sequence annotations. Nucleic Acids Res 47:D351–D360. 10.1093/nar/gky1100. 30398656 PMC6323941

[56] Eddy SR. 2011. Accelerated profile HMM searches. PLoS Comput Biol 7:e1002195. 10.1371/journal.pcbi.1002195. 22039361 PMC3197634

