# Supplementary material for: Genomic Characterization and Antimicrobial Susceptibility of Dromedary-Associated Staphylococcaceae from the Horn of Africa
Source: Appl Environ Microbiol. 2022 Oct 13;88(21):e01146-22. doi: 10.1128/aem.01146-22 (PMC9642023; doi:10.1128/aem.01146-22)
Supplement: Supplemental file 1 — Fig. S1 to S4. Download aem.01146-22-s0001.pdf, PDF file, 9.9 MB [file aem.01146-22-s0001.pdf]

Figure S1A *Mammaliicoccus sciuri*

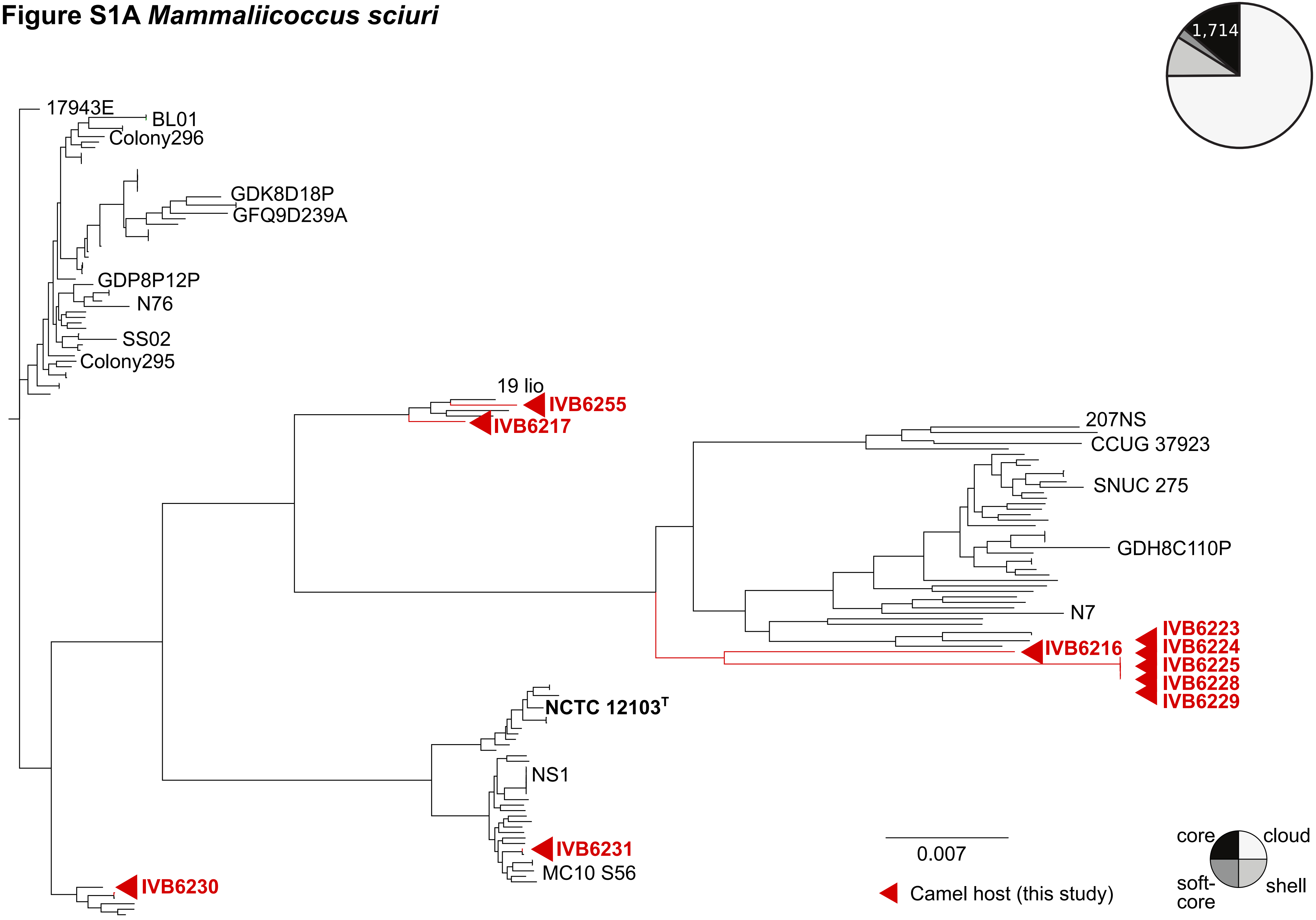

Figure S1B *Staphylococcus agnetis*

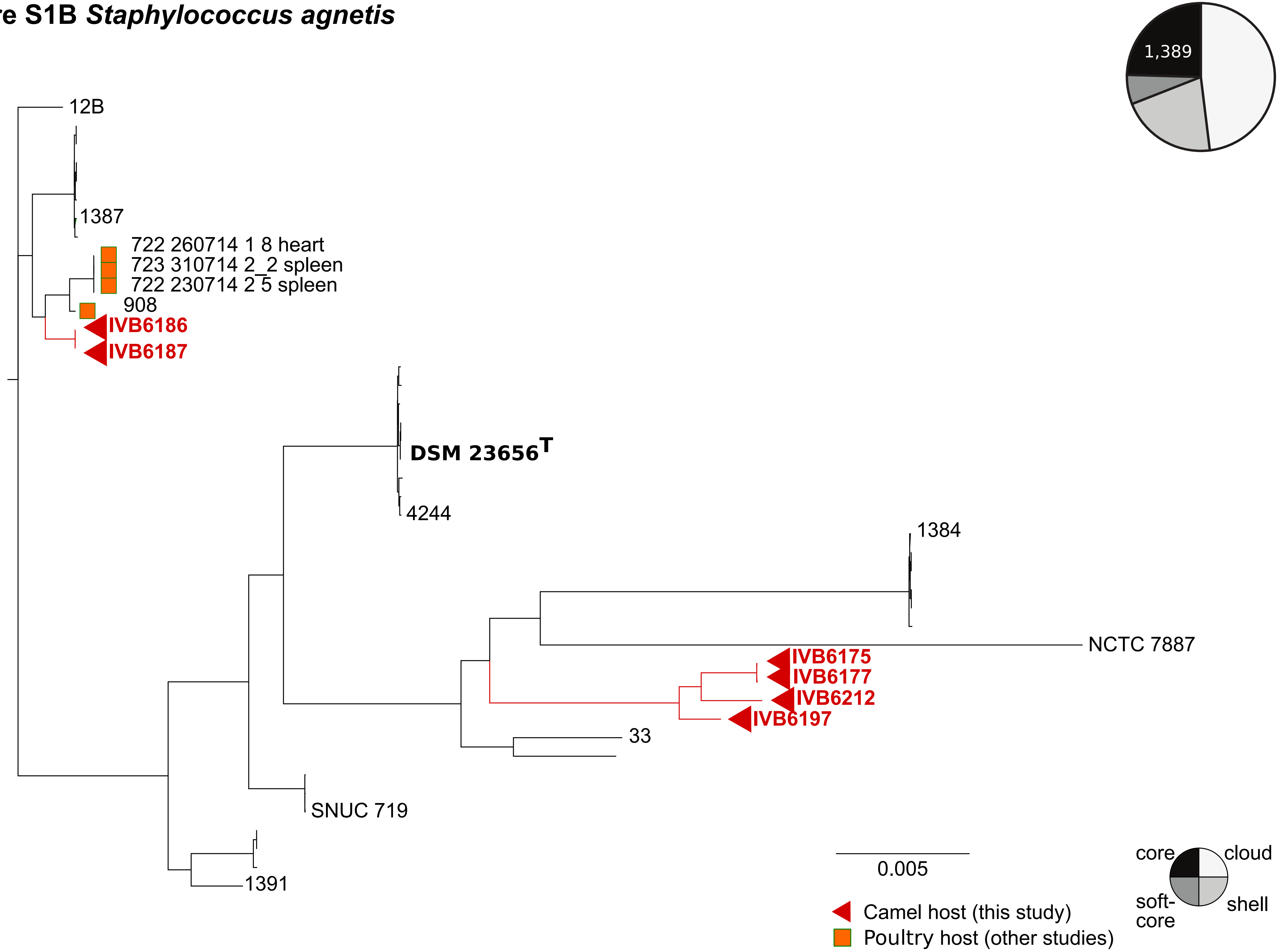

Figure S1C *Staphylococcus aureus*

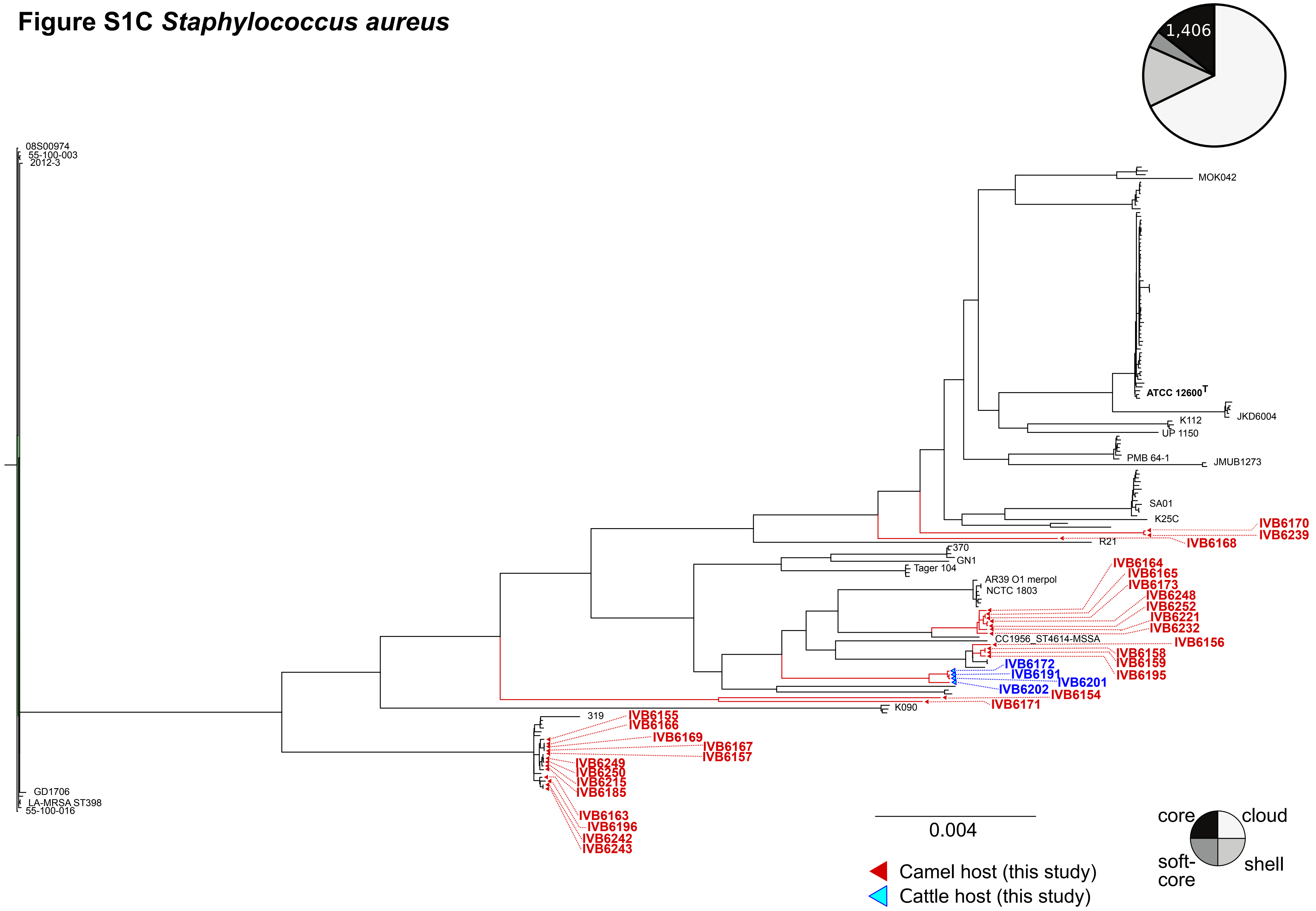

Figure S1D *Staphylococcus delphini*

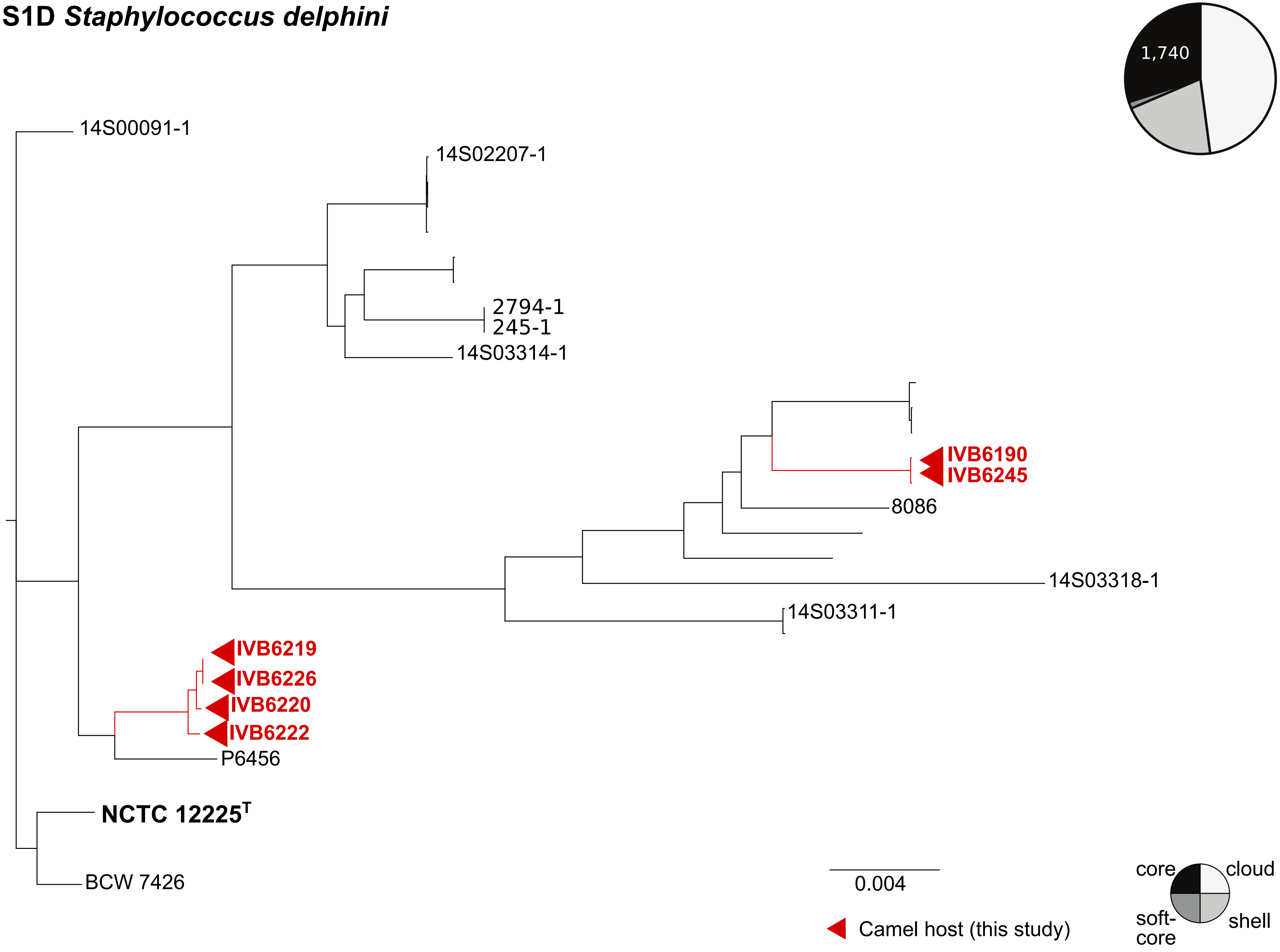

Figure S1E *Staphylococcus epidermidis*

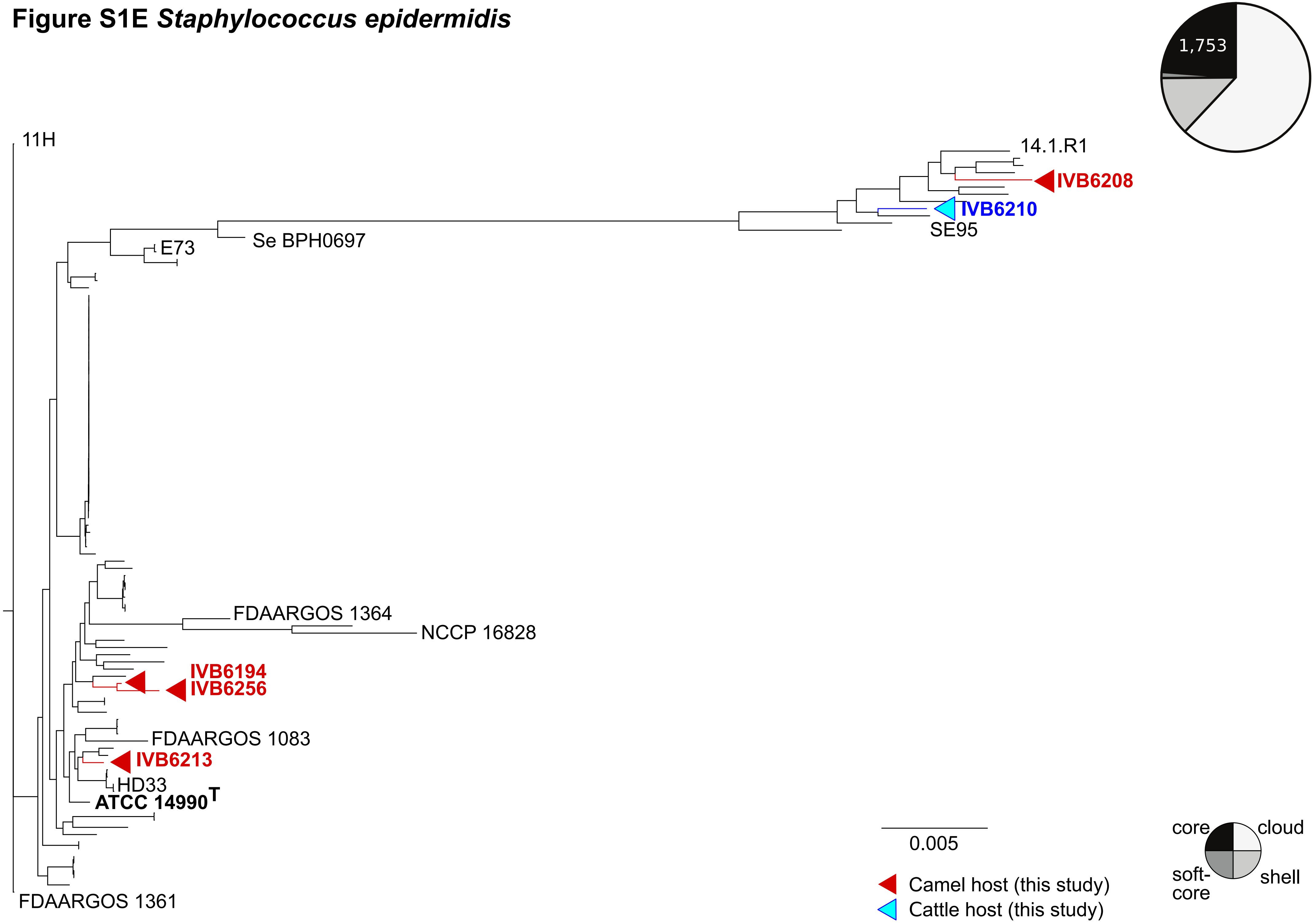

Figure S1F *Staphylococcus simulans*

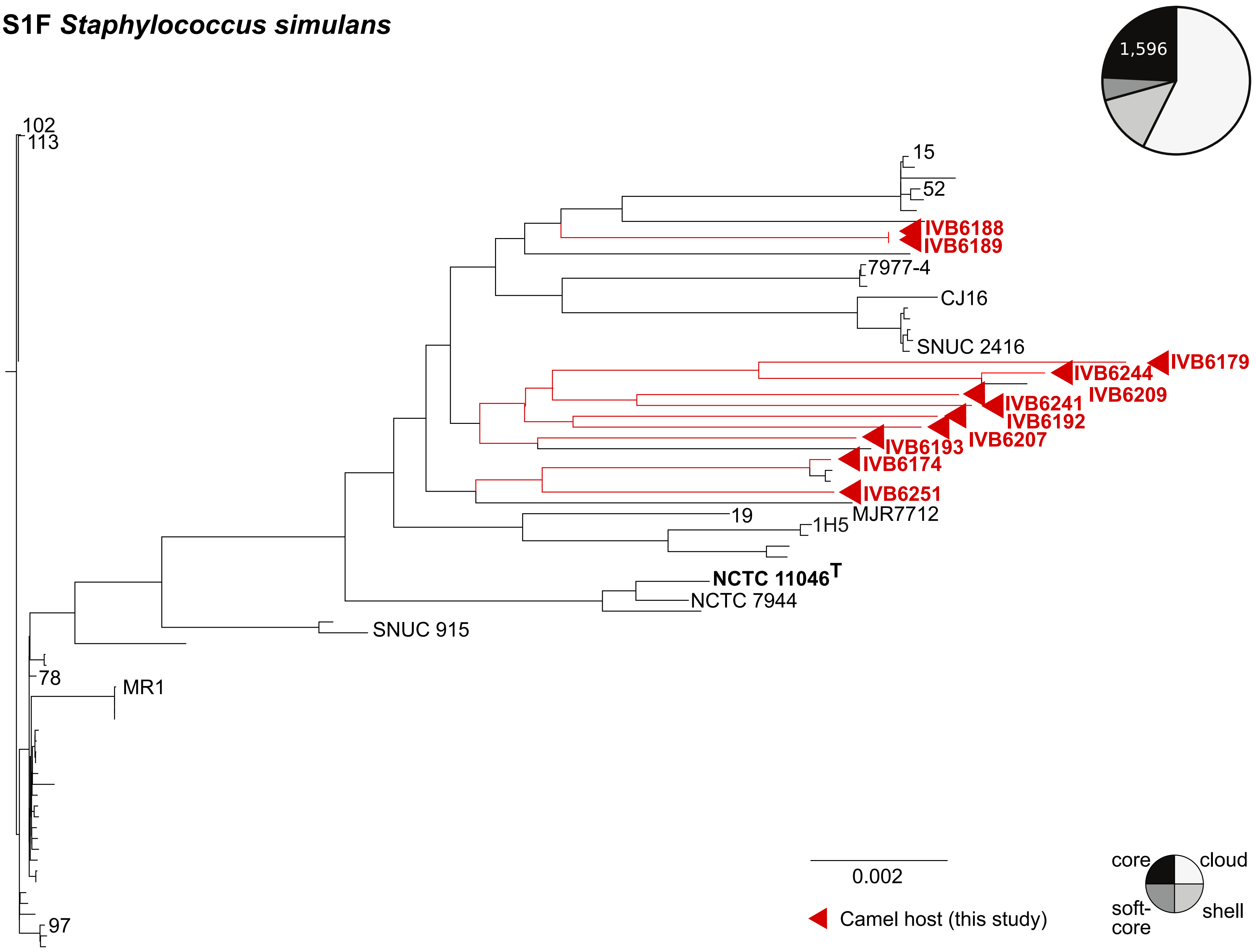

Figure S1G *Staphylococcus arlettae*

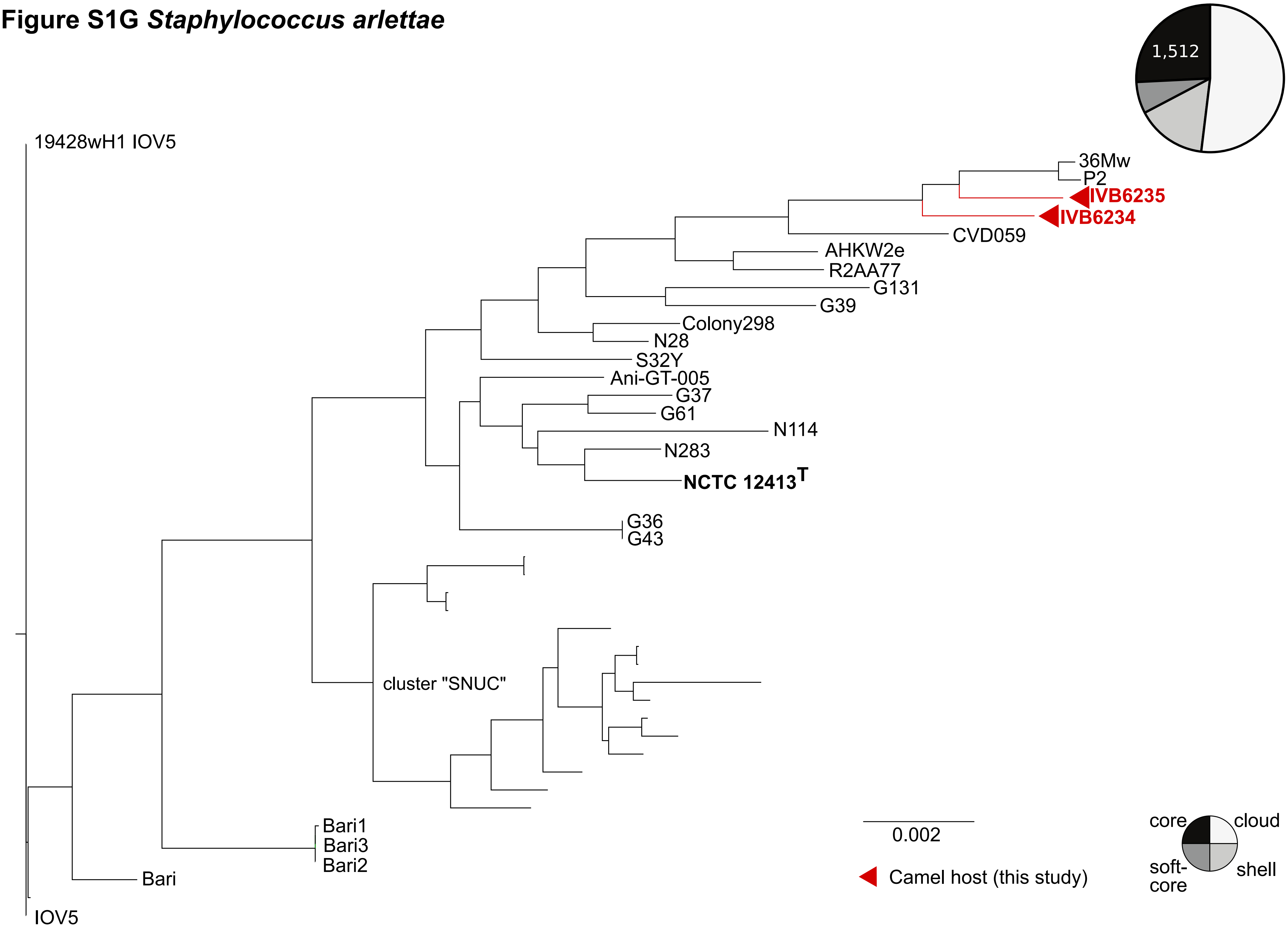

Figure S1H *Staphylococcus chromogenes*

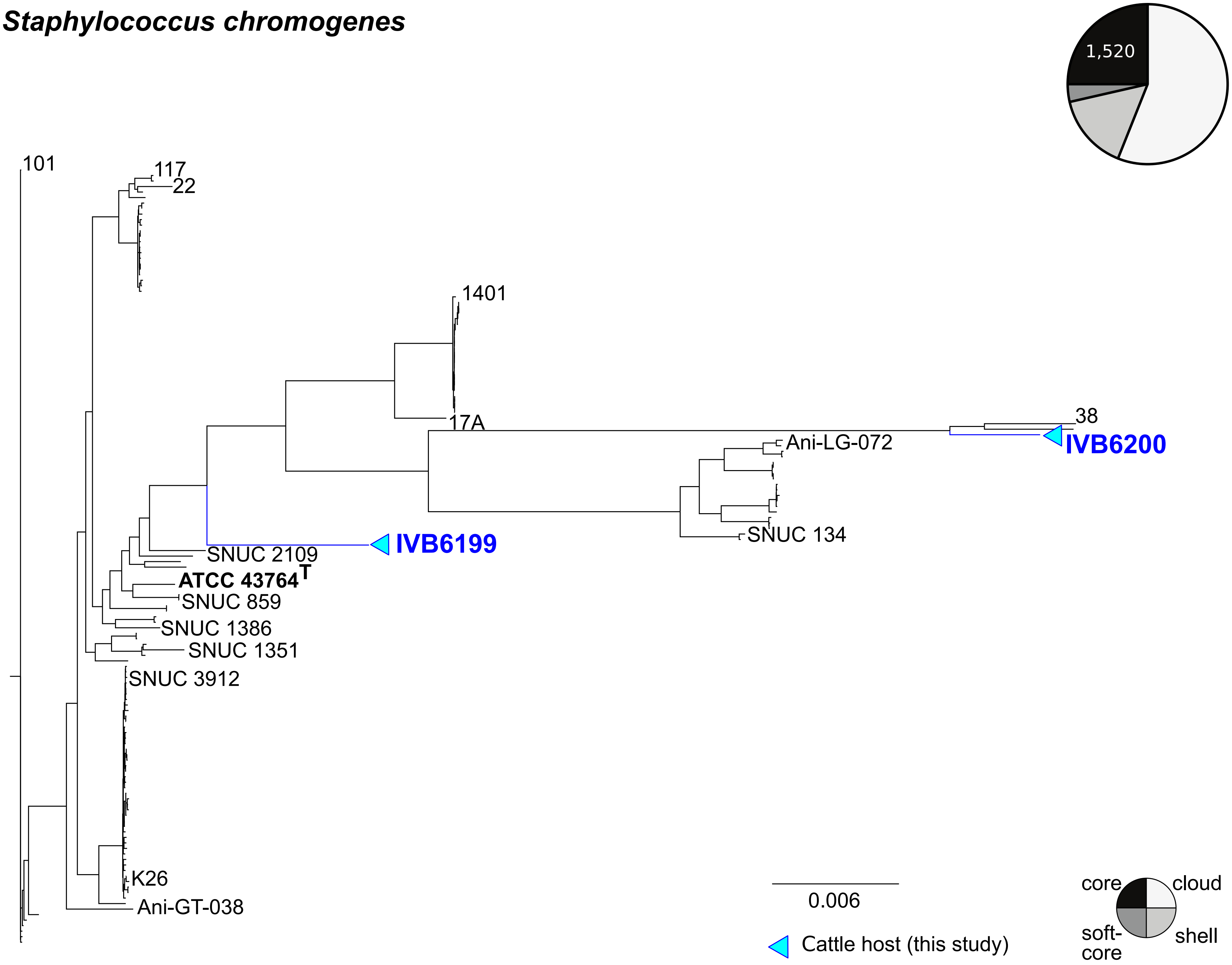

Figure S1I *Staphylococcus felis*

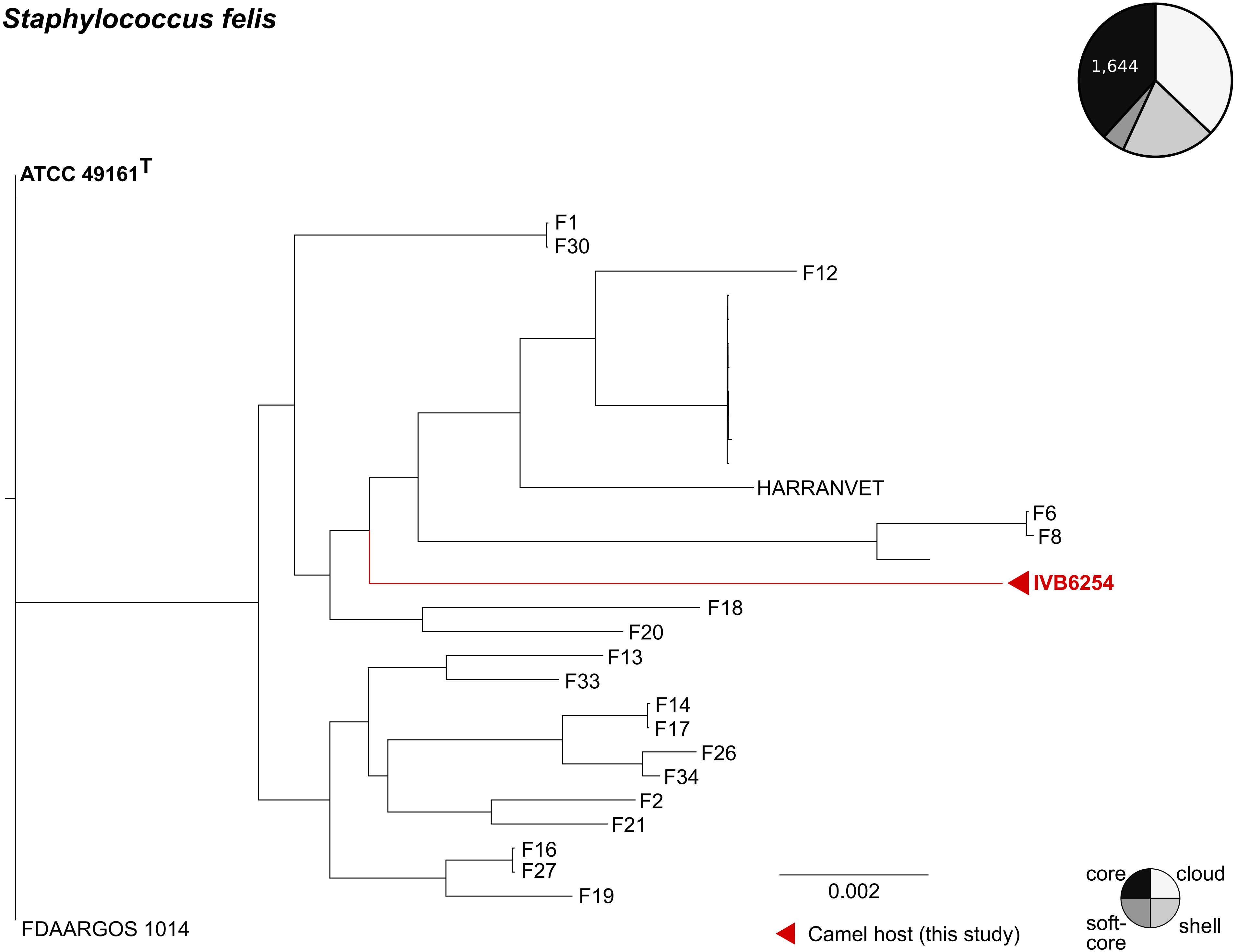

Figure S1J *Staphylococcus hominis*

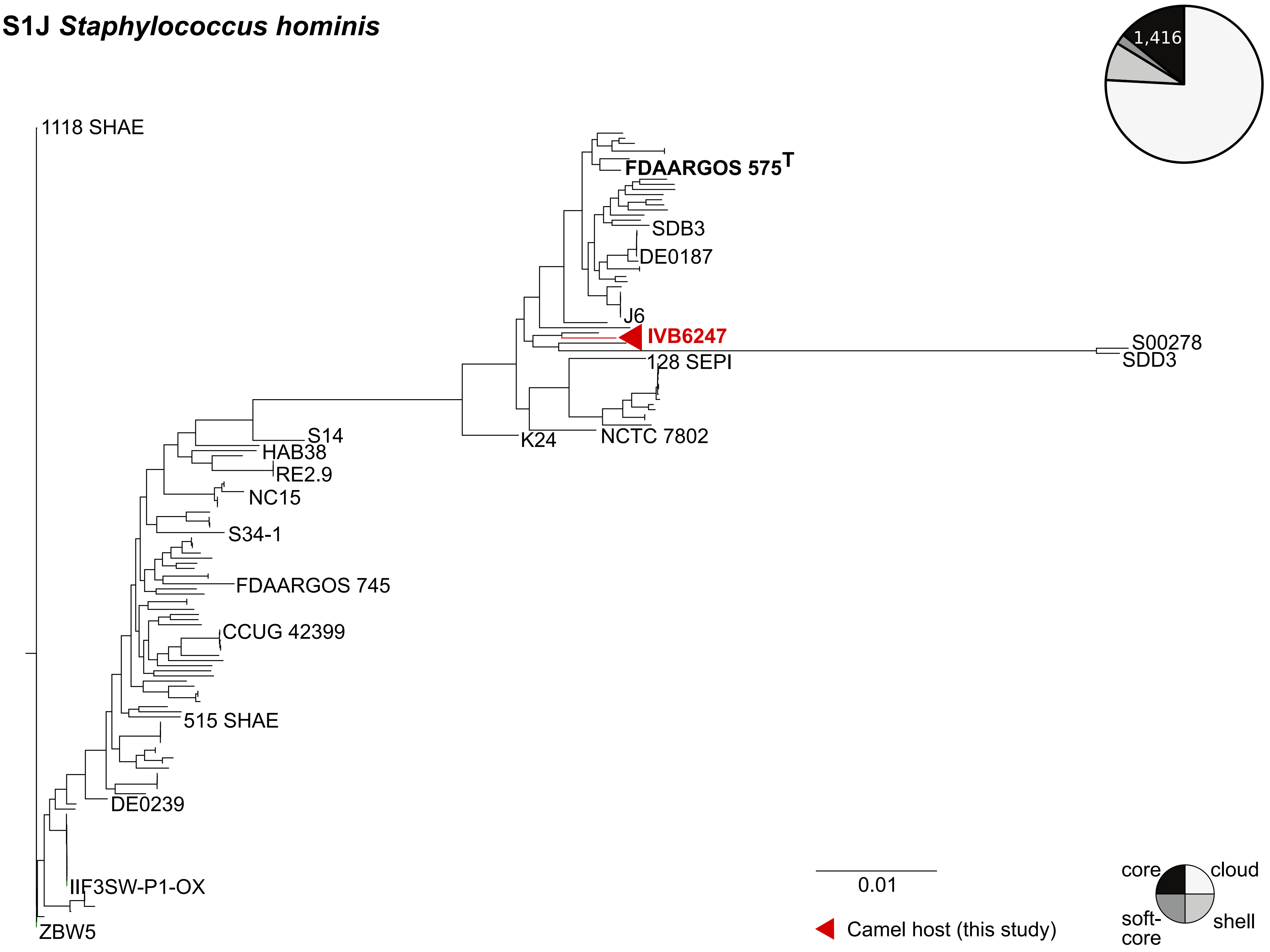

Figure S1K *Staphylococcus pasteuri*

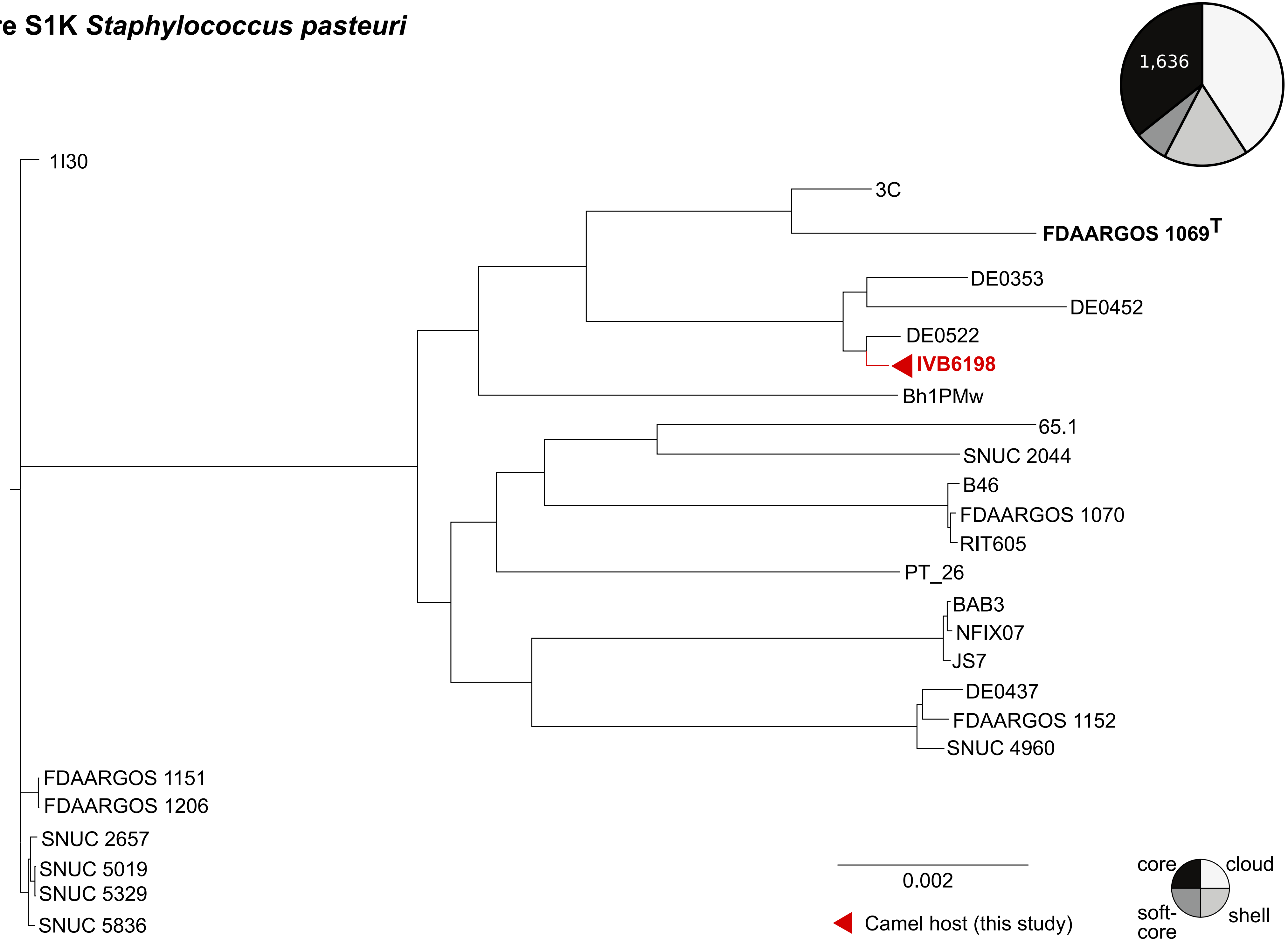

Figure S1L *Staphylococcus schleiferi*

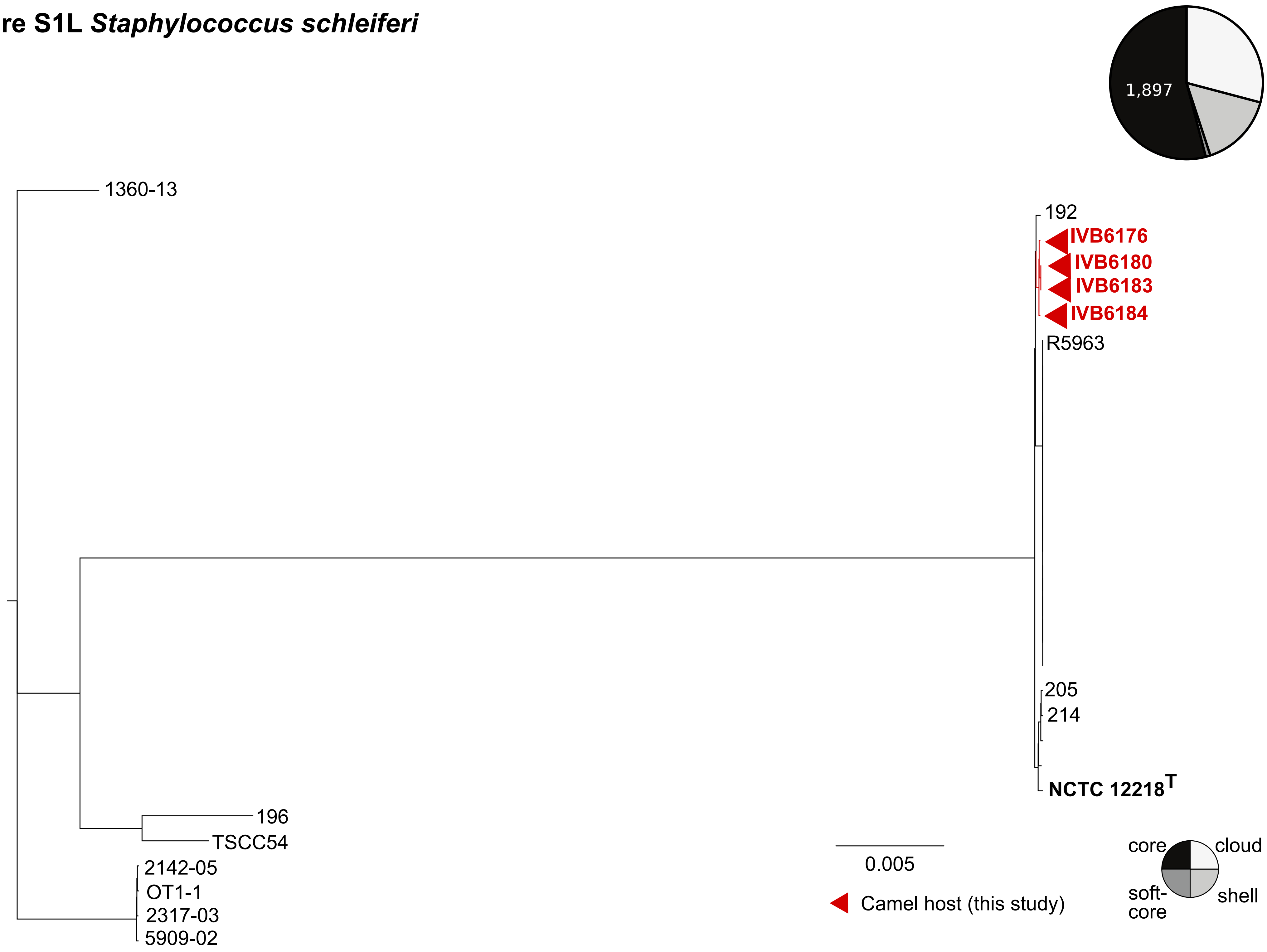

Figure S1M *Staphylococcus ureilyticus*

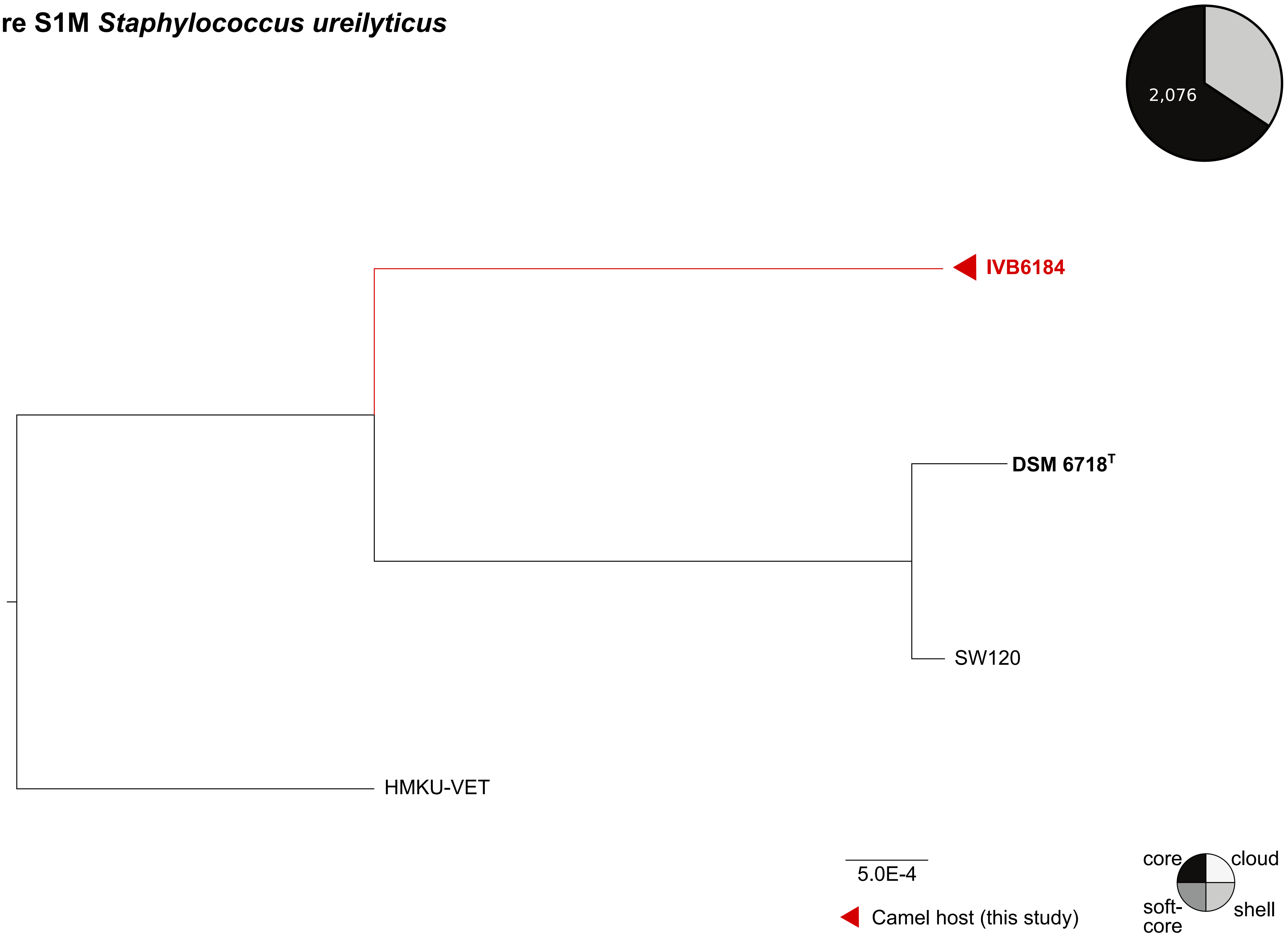

Figure S1N *Staphylococcus muscae*-like

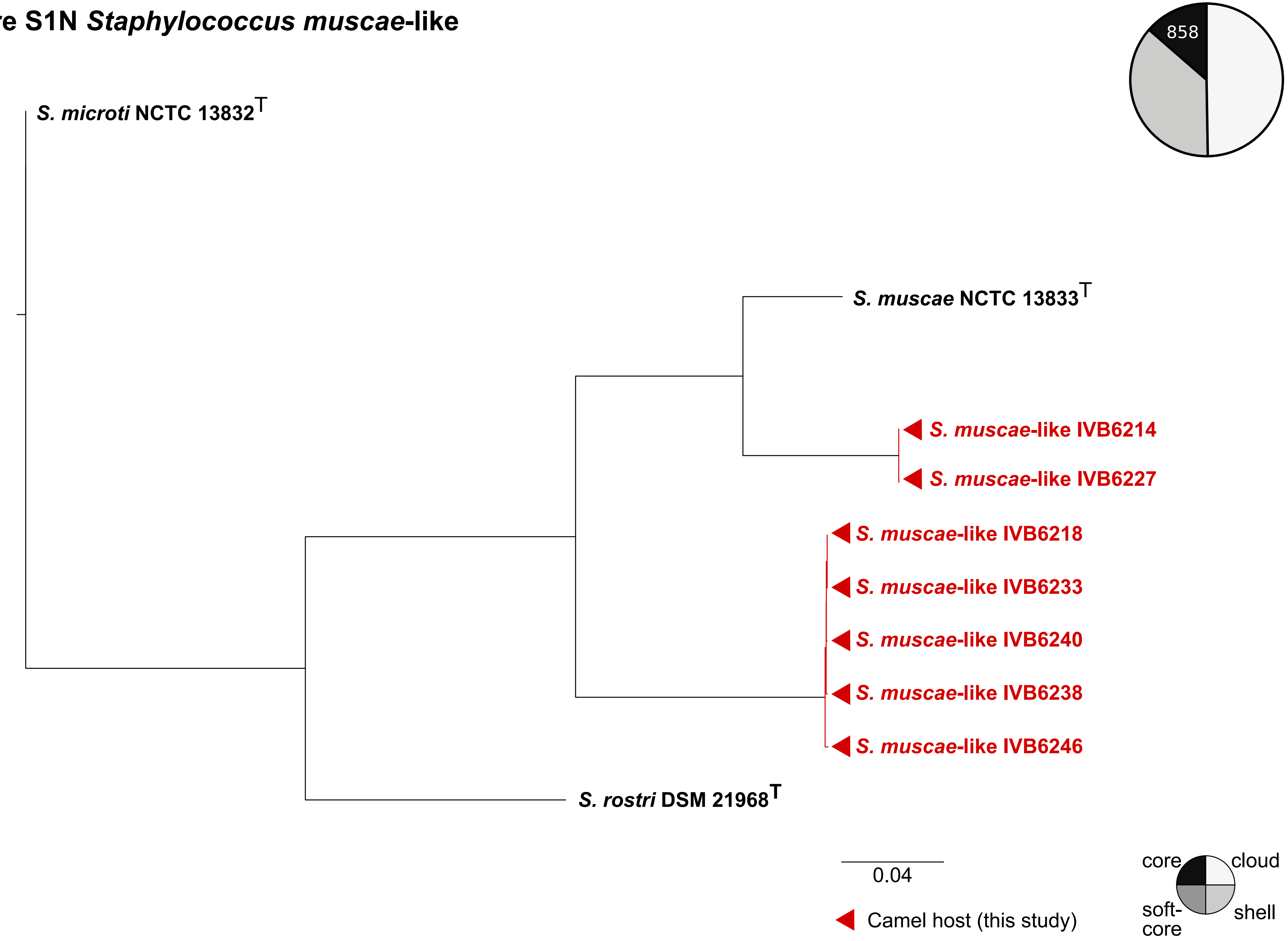

Figure S1O *Staphylococcus aureus*

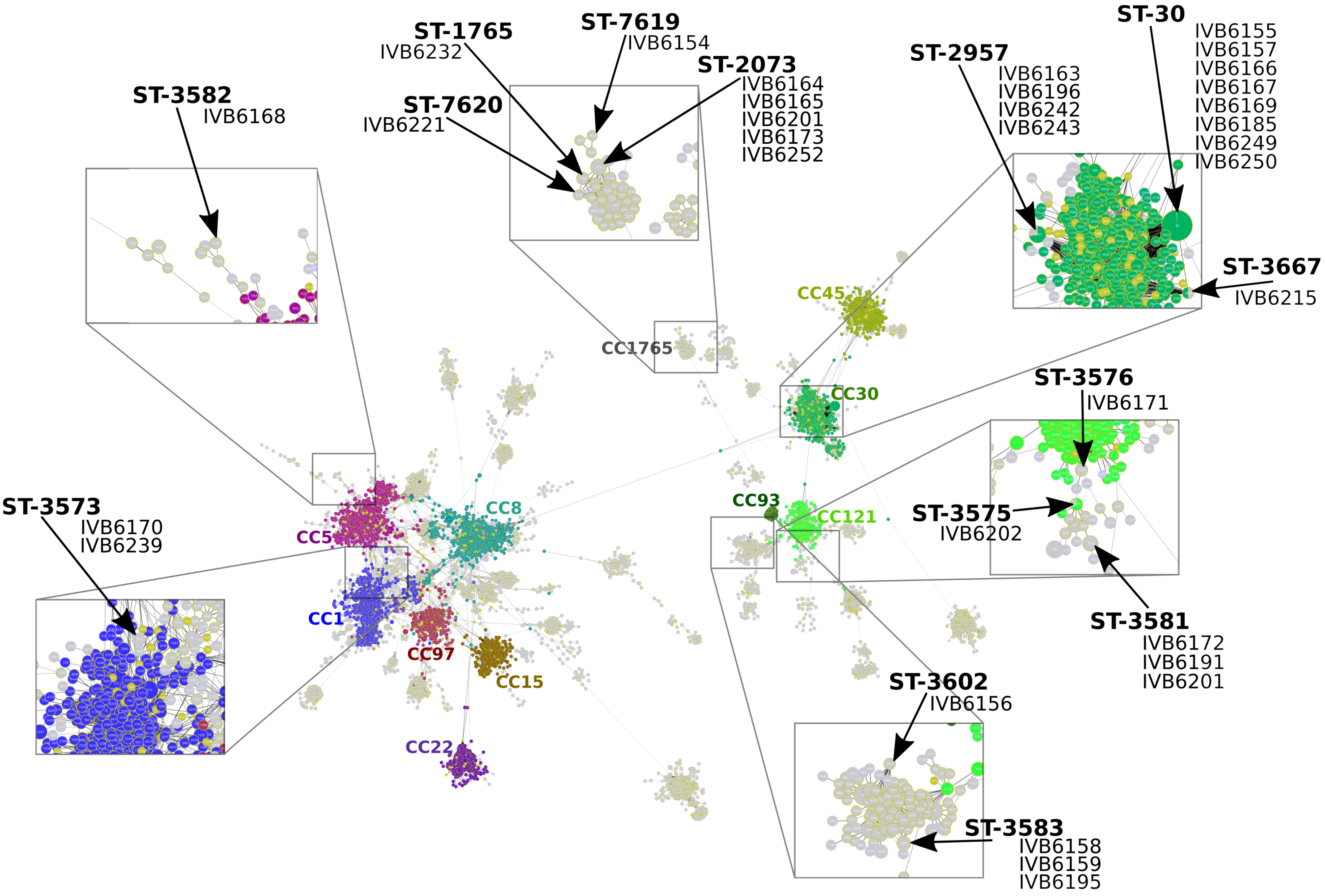

Figure S1P *Staphylococcus epidermidis*

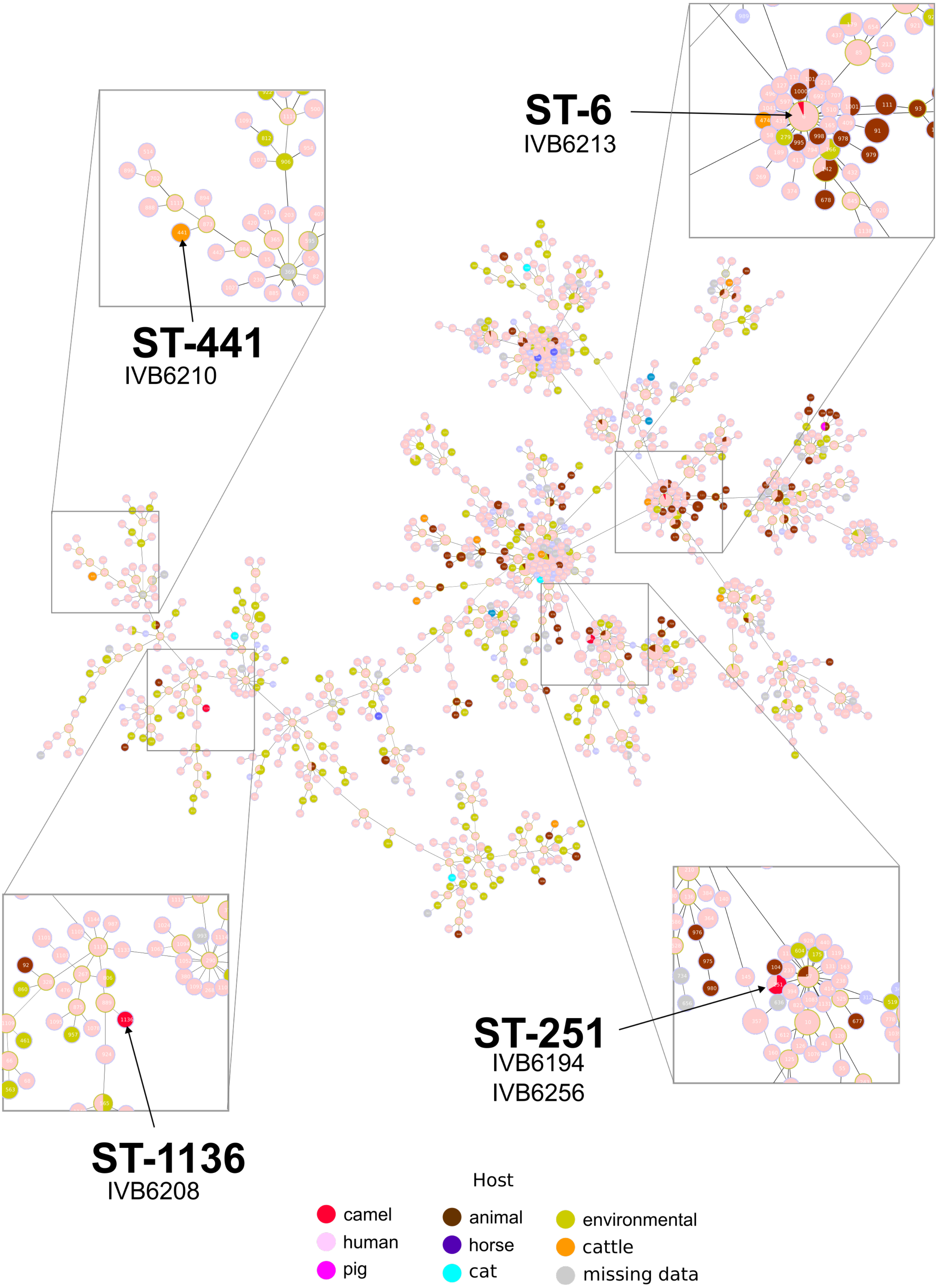

Figure S1Q *Mammaliicoccus sciuri*

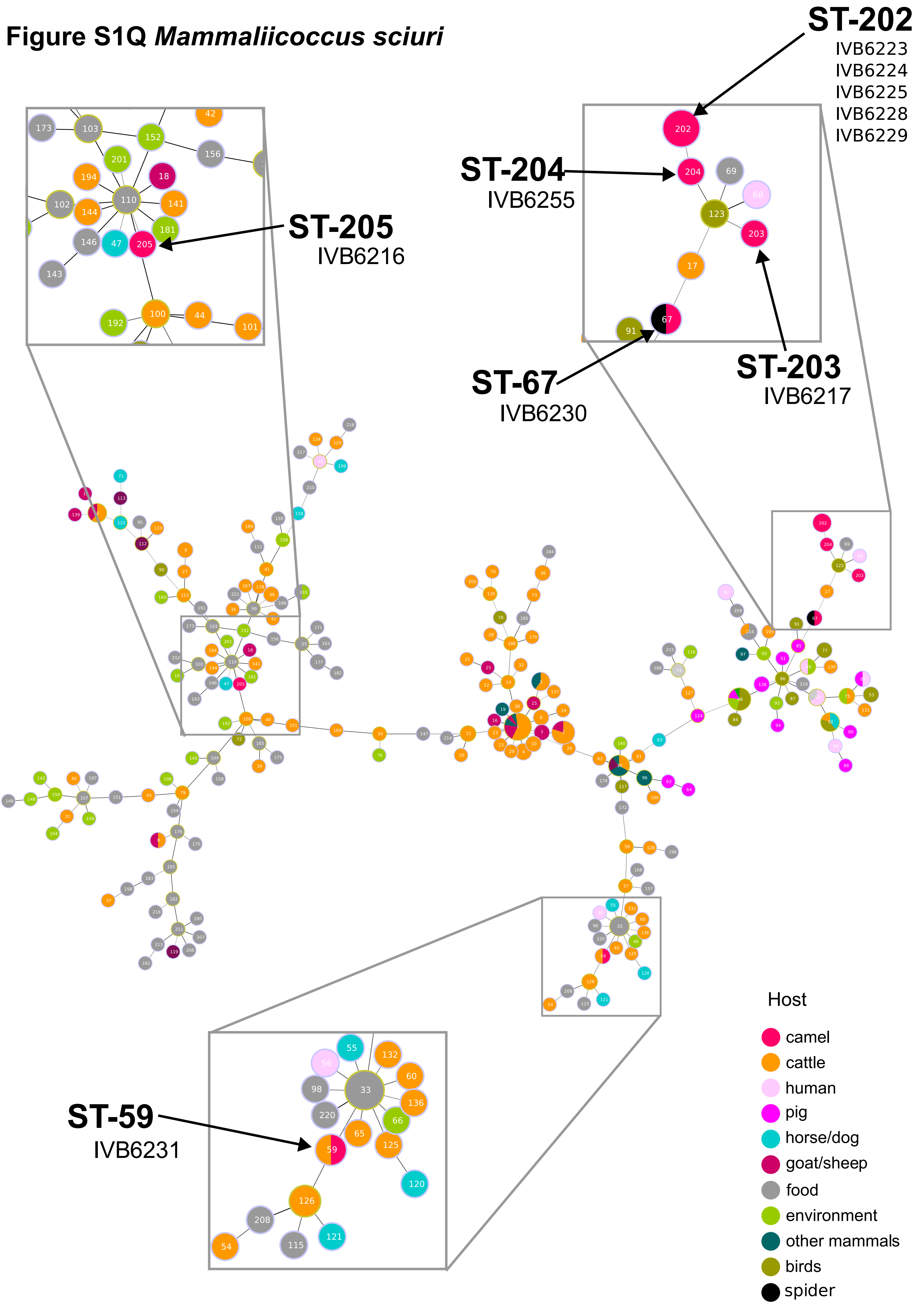

Figure S1R *Staphylococcus hominis*

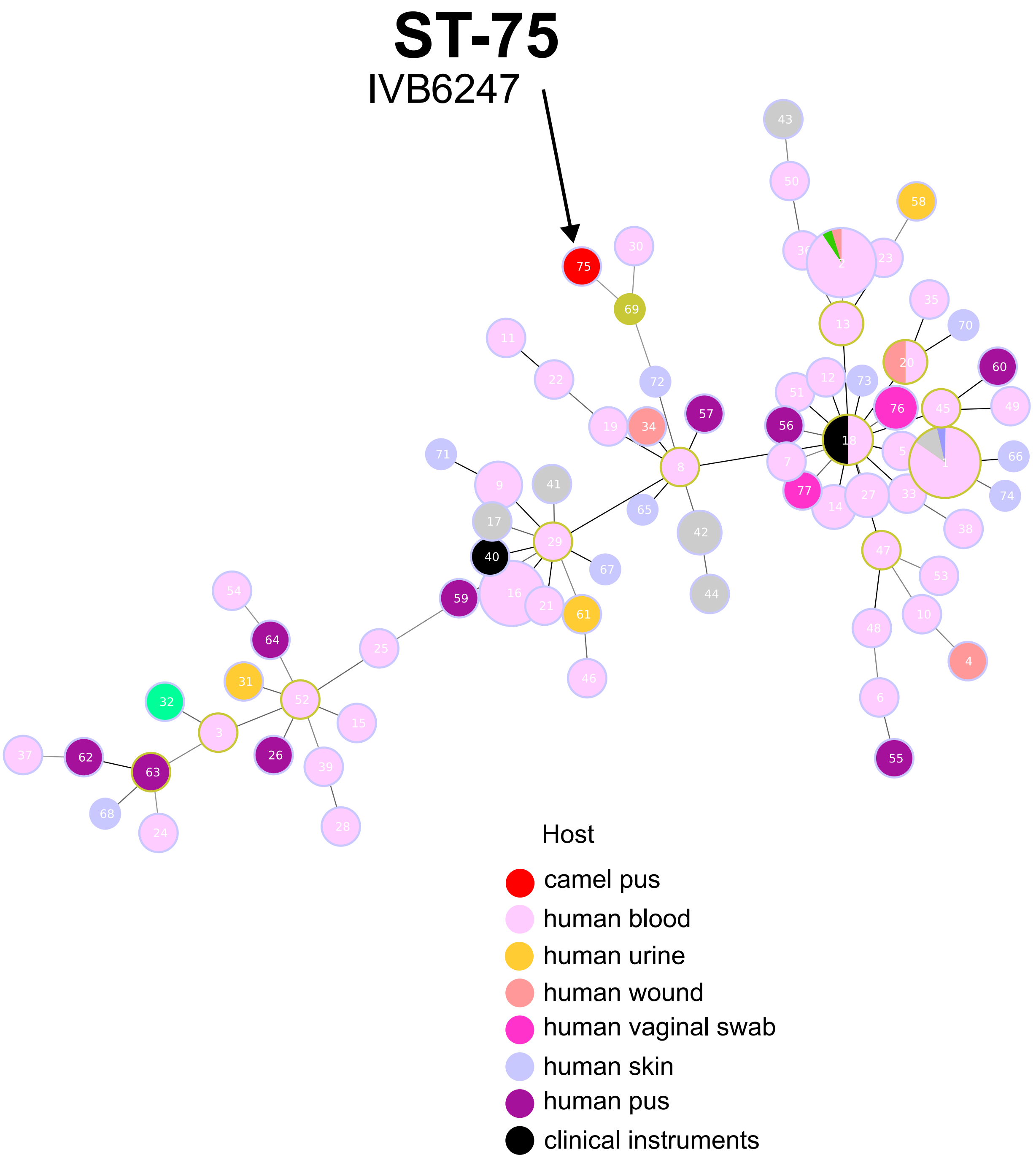

Figure S1S *Staphylococcus chromogenes*

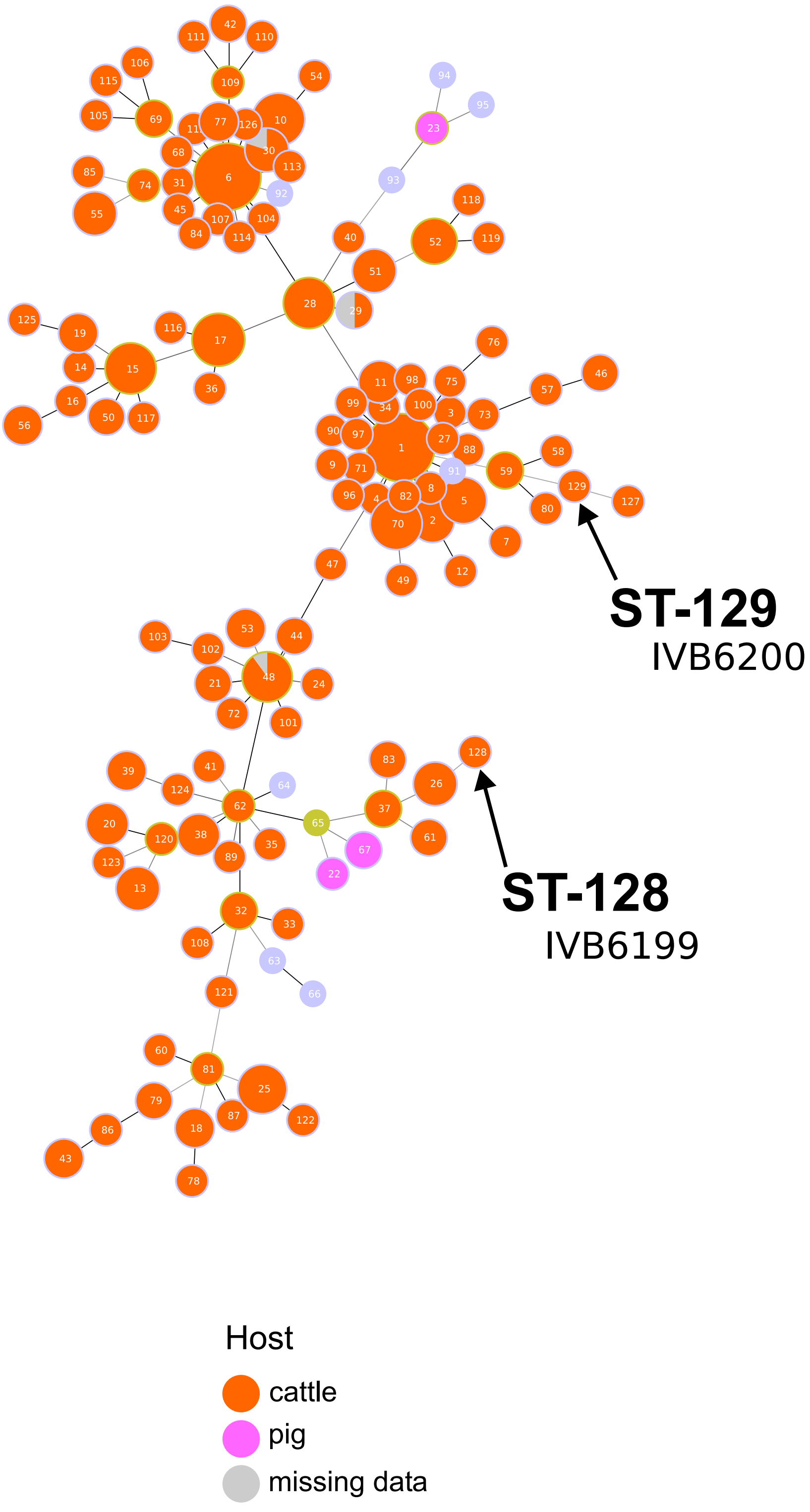

Figure S1. Core genome-based phylogenetic trees and minimum spanning trees of *Staphylococcaceae*. (A-N) The *Staphylococcus* phylogenetic trees for each species are provided. For a given species tree, all the corresponding strains from this study were included, along with reference whole genome sequences retrieved from GeneBank. For readability, only complete genomes for *S. aureus* and *S. epidermidis* were downloaded and used in the corresponding species phylotrees. For the NCBI *S. aureus*, since the dataset of complete genomes was still too large (>800), only a subset of the complete genomes was used: all the N=95 non-human genomes at complete or chromosome assembly levels, plus N=47 randomly picked human *S. aureus* genomes (at complete or chromosome assembly levels). Core genomes and alignments were built with Roary and MAFFT, the phylotrees were constructed with IQ-TREE2 and its PhyML option. The camel strains of our dataset are shown with red arrows, while the cattle strains of this study are indicated by cyan arrows. For each species panel, a pie chart overview of the pan and core distribution is also provided, showing the size of the core genome used to build the corresponding phylotree. **(O-S)** Minimum Spanning Tree (MST) based on multilocus sequence typing (MLST) data. The strain data were downloaded from PubMLST and the trees were built with the goeBURST algorithm in Phyloviz 2.0. **(O) MST of *S. aureus* Sequence Types (STs), including camel strains of this study.** A total of 37,357 strains were thereby included. Zoom-ins from the underlying full tree are shown to visualize the context of East African camel strains of this study. **(P) MST of *S. epidermidis* including the camel strains of this study.** A total of 1,785 strains were thereby included. Zoom-ins from the underlying full tree are shown to visualize the location of the strains of this study. **(Q) MST of *M. sciuri* including the camel strains of this study.** A total of 337 strains were thereby included. **(R) MST of *S. hominis* including the camel strains of this study.** A total of 137 strains were thereby included. **(S) MST of *S. chromogenes* including the bovine strains of this study.** A total of 370 strains were thereby included.

Figure S2A *S. aureus*

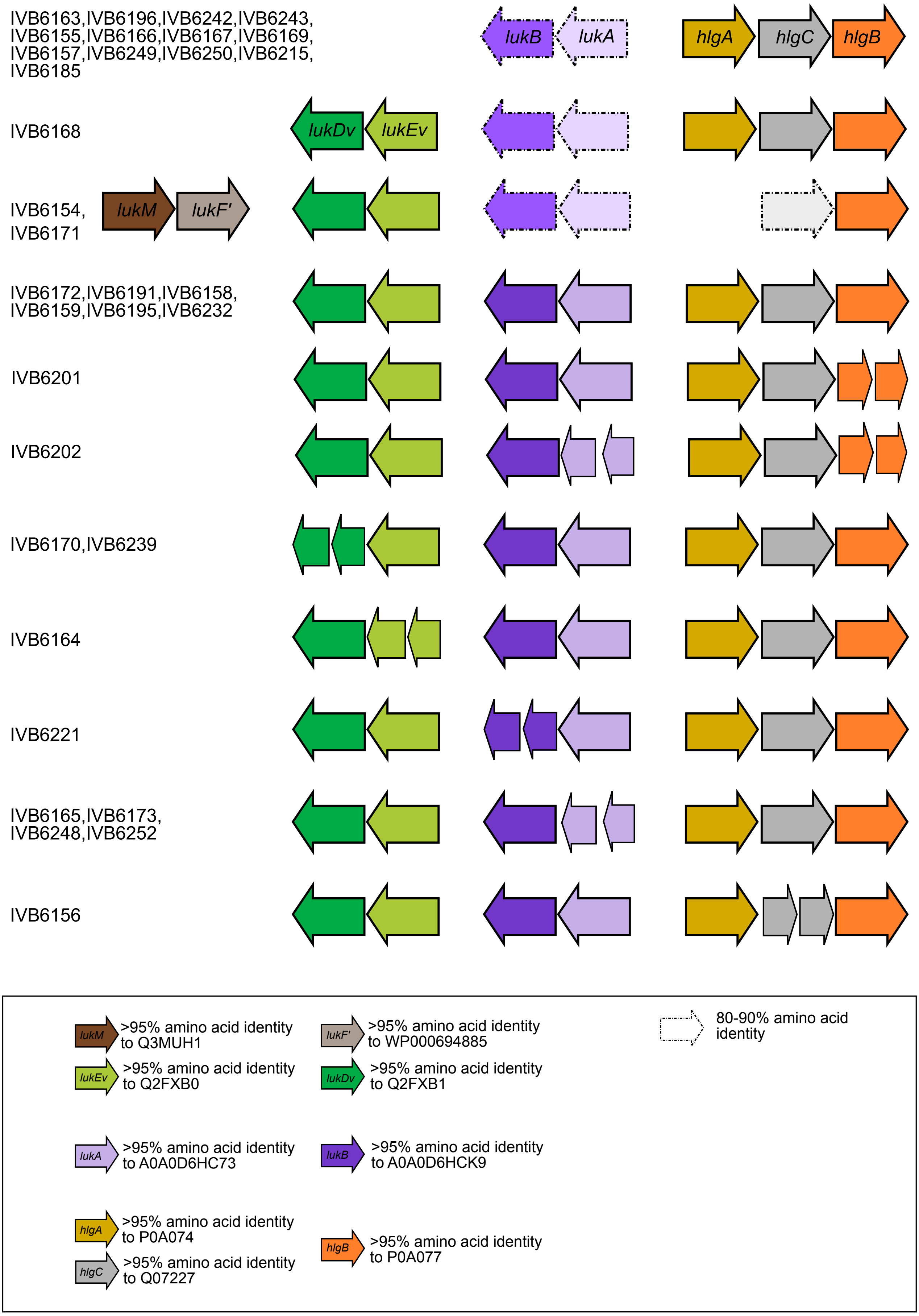

Figure S2B *S. aureus*

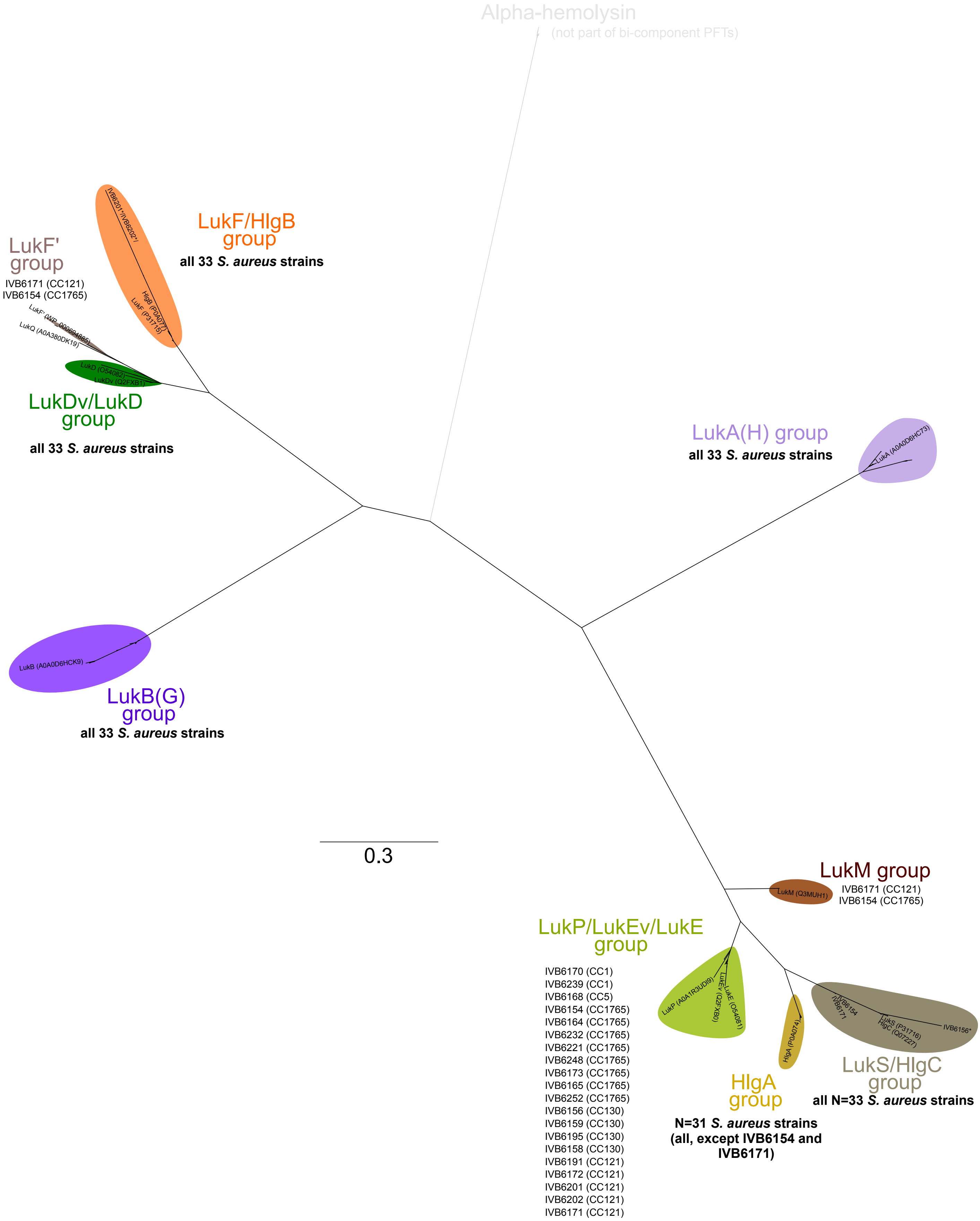

*second cds

Figure S2C *S. schleiferi*

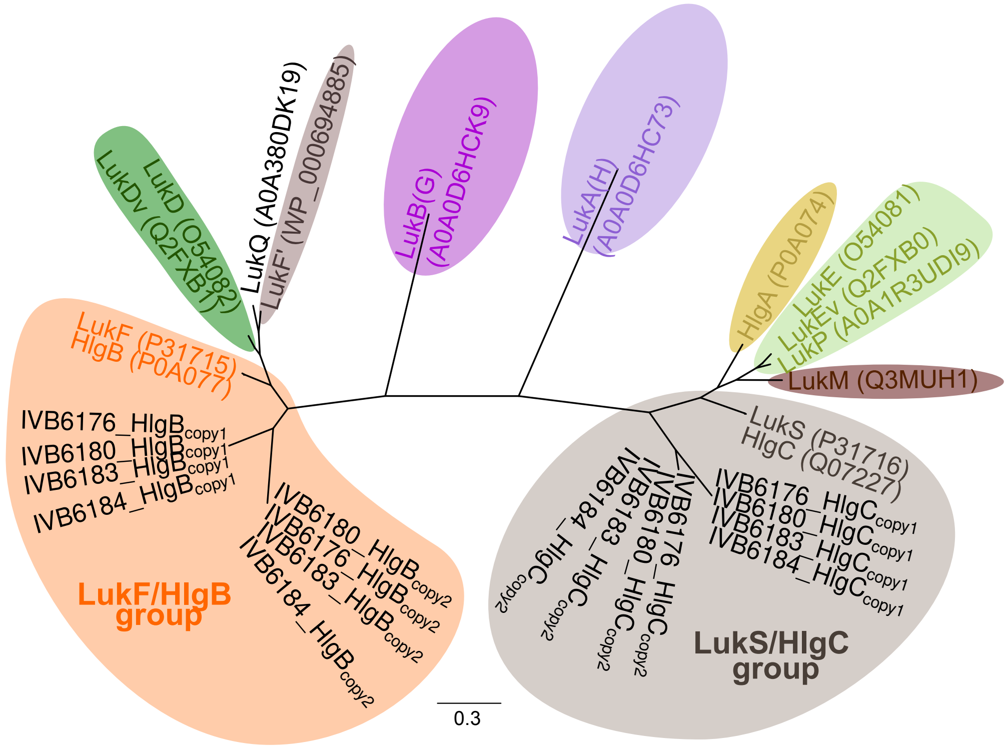

Figure S2D

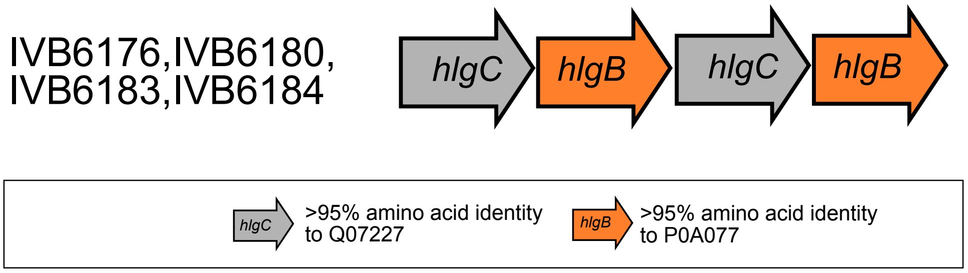

Figure S2E

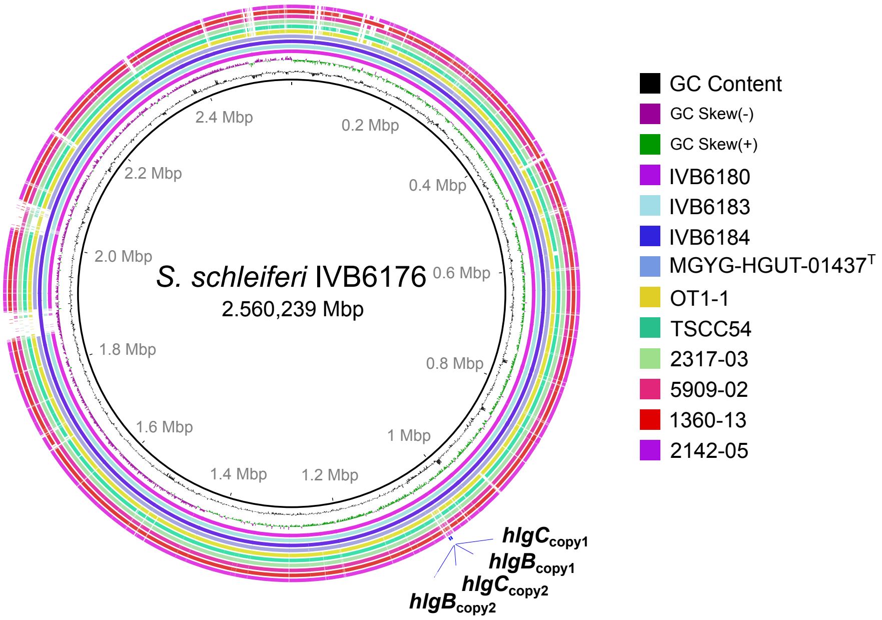

Figure S2F *S. delphini*

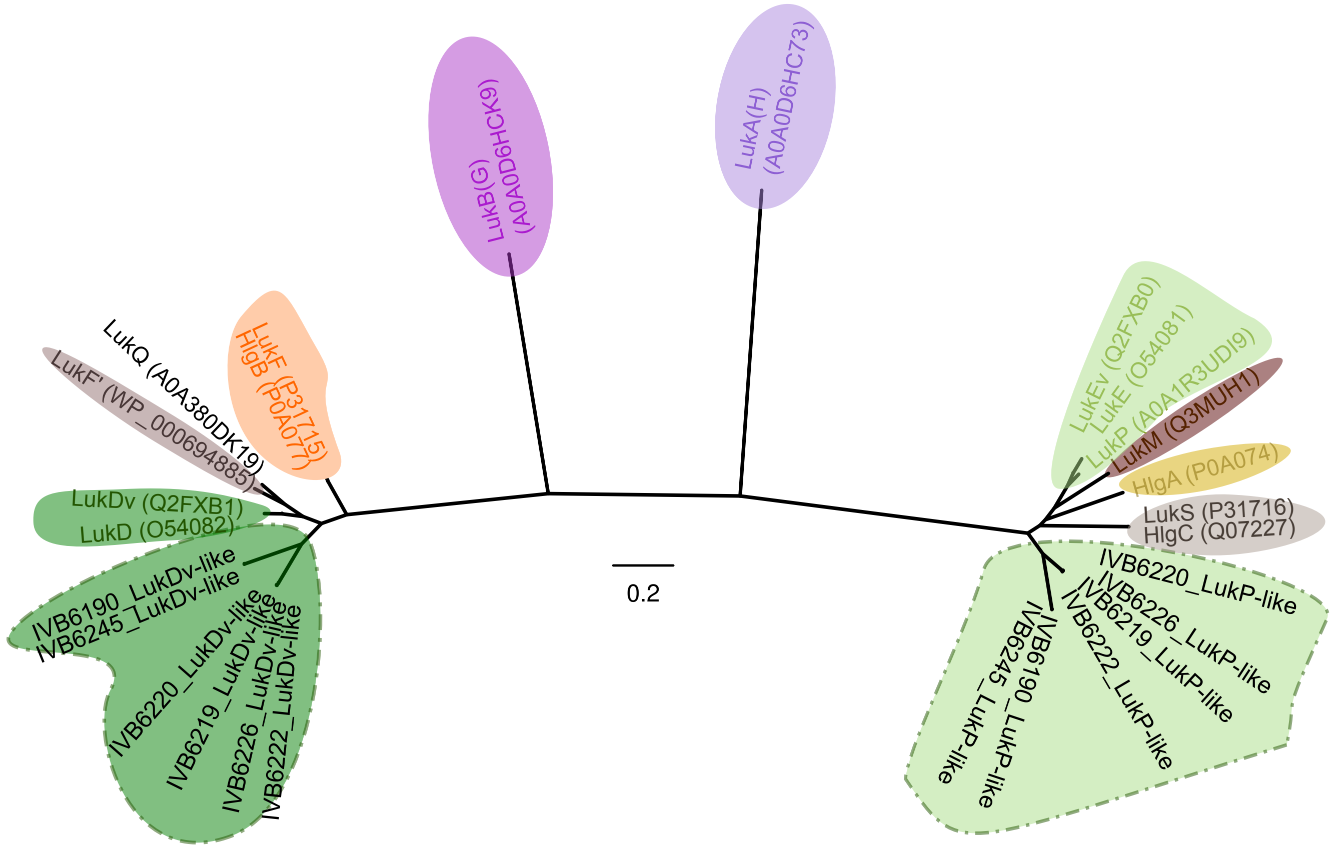

Figure S2G

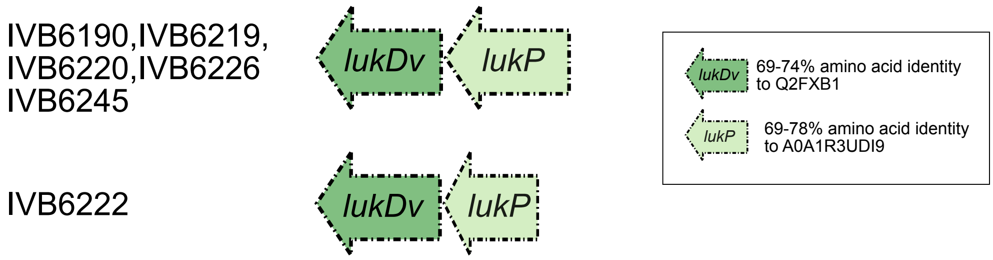

Figure S2H

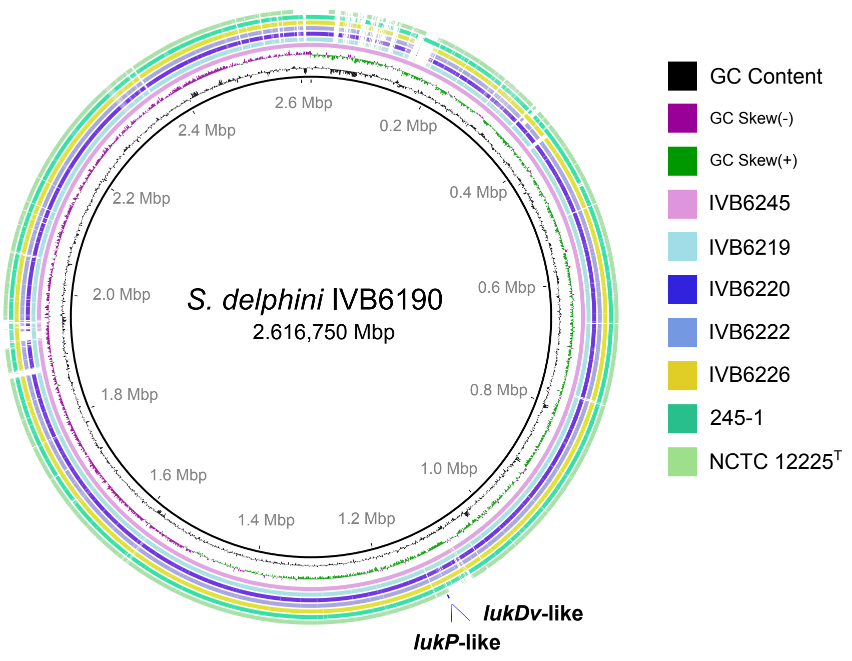

Figure S2. Presence of bi-component leukocidin-encoding genes in *S. aureus*, *S. schleiferi* and *S. delphini* as well their phylogenetic relationship in comparison to reference sequences. The phylogenetic trees were calculated using amino acid sequences aligned with MAFFT and IQ-TREE2 and plotted with FigTree. **(A) PFT genes organisation in *S. aureus* of this dataset. (B) Phylotree from PFT proteins in *S. aureus*. (C) Phylotree from PFT proteins in *S. schleiferi*. (D) PFT genes organisation in *S. schleiferi* of this dataset. (E) Genomic location and presence/absence of PFT genes in *S. schleiferi* strains with BRIG. (F) Phylotree from PFT proteins in *S. delphini*. (G) PFT genes organisation in *S. delphini* of this dataset. (H) Genomic location and presence/absence of PFT genes in *S. delphini* strains with BRIG.**

Figure S3A

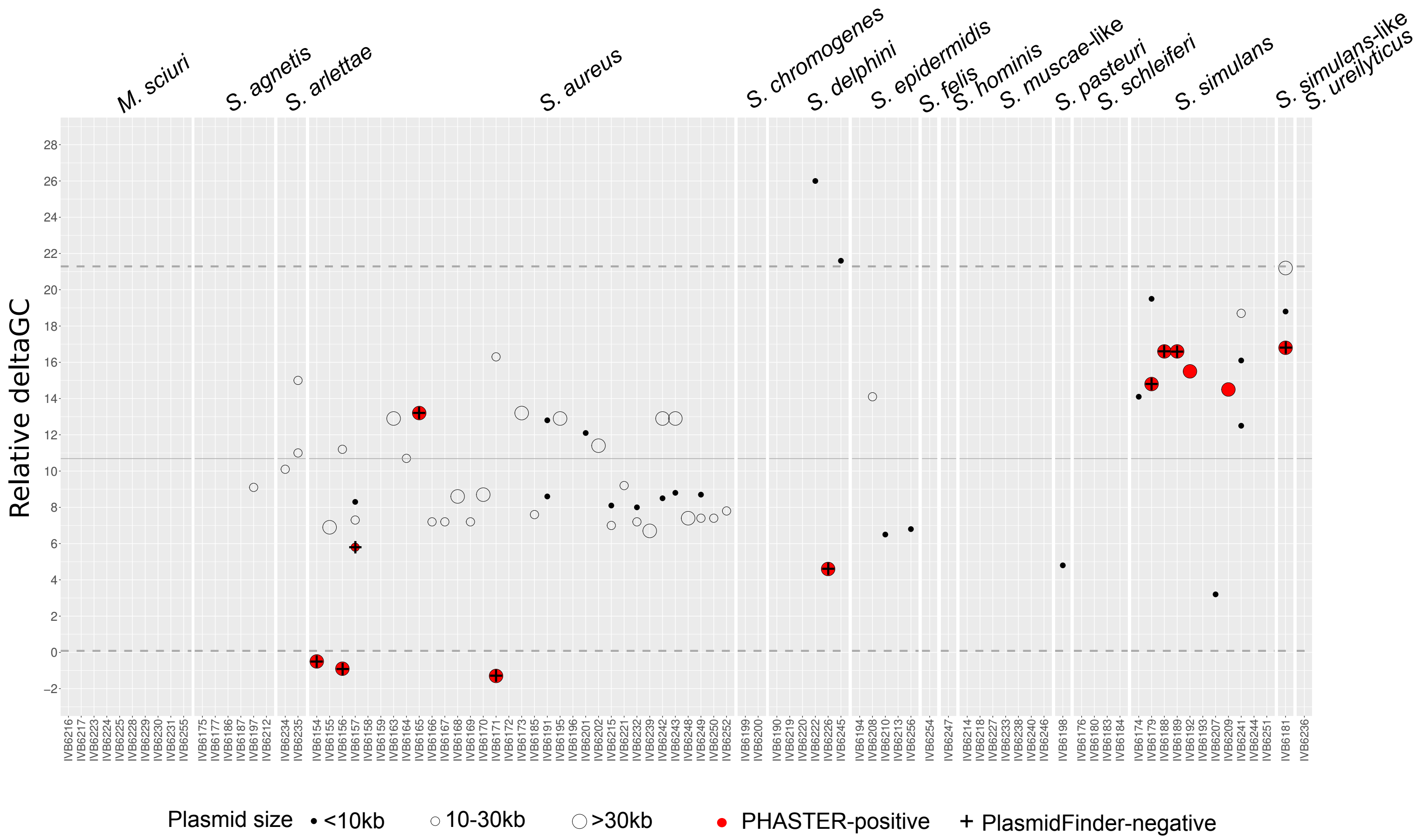

Figure S3B

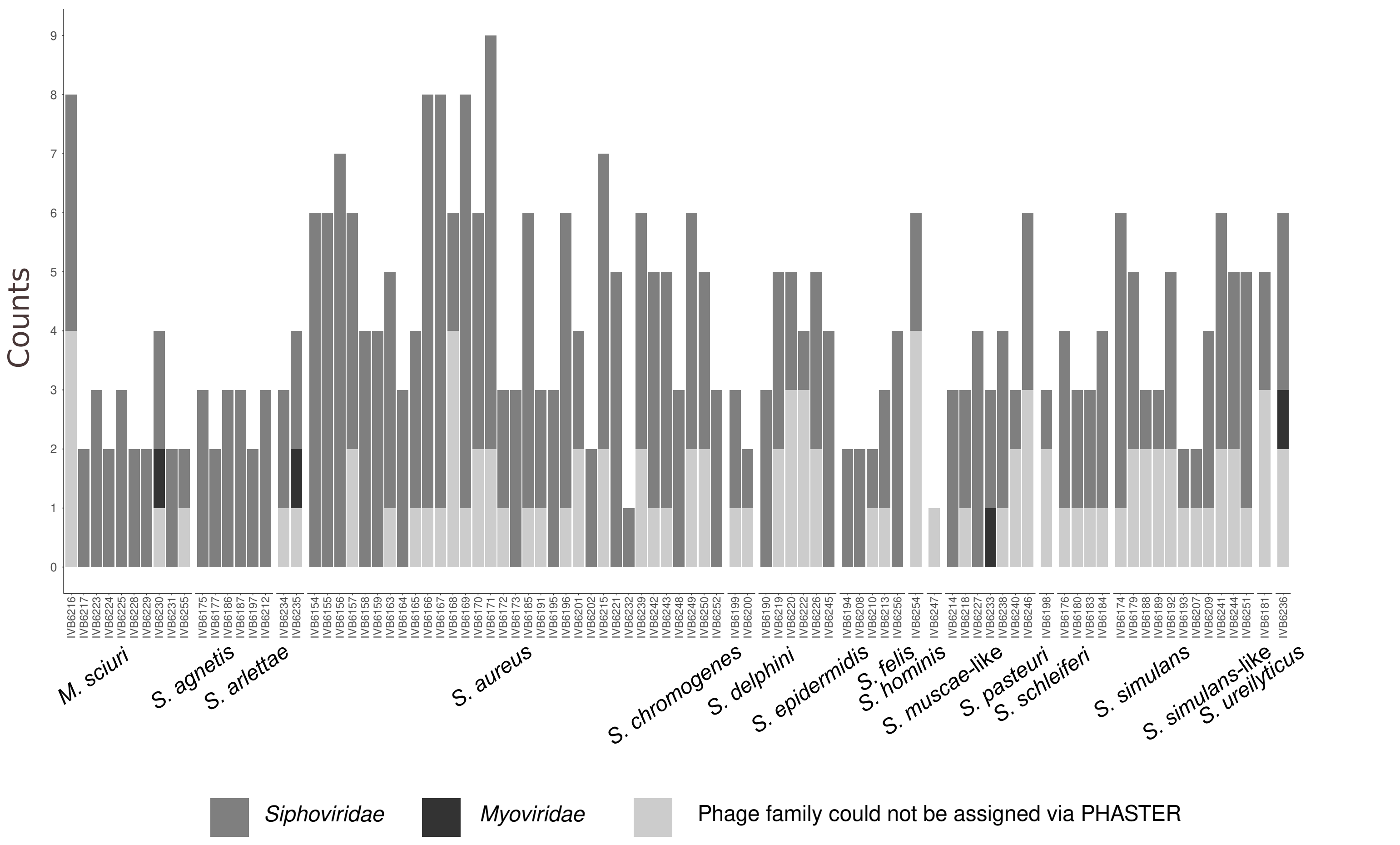

Figure S3. Mobilome characterization of the *Staphylococcaceae* dataset. (A) Overview of plasmid presence across the strains and the relative GC content differences between bacterial host chromosomes and the plasmids. Each dot represents a circularized non chromosomal DNA identified as a plasmid by PlasmidFinder. The mean relative deltaGC value is shown by a continuous grey line, while the two dashed grey lines correspond to two standard deviation (2*SD) from the relative deltaGC mean value. Three PlasmidFinder positive hits (*S. delphini* IVB6222, *S. delphini* IVB6245 and *S. simulans*-like IVB6181) had a relative deltaGC value beyond the threshold. Three PlasmidFinder positive hits (*S. aureus* IVB6165, *S. simulans* IVB6192 and *S. simulans* IVB6209) were also identified as prophages by PHASTER. PlasmidFinder-negative plasmids are marked with an asterisk **(B) Taxonomic family distribution of prophages identified by PHASTER.** It includes the intact (score >90), questionable (score 70-90) and incomplete (score <70) putative prophage hits according to the pipeline's scoring method.

Figure S4A

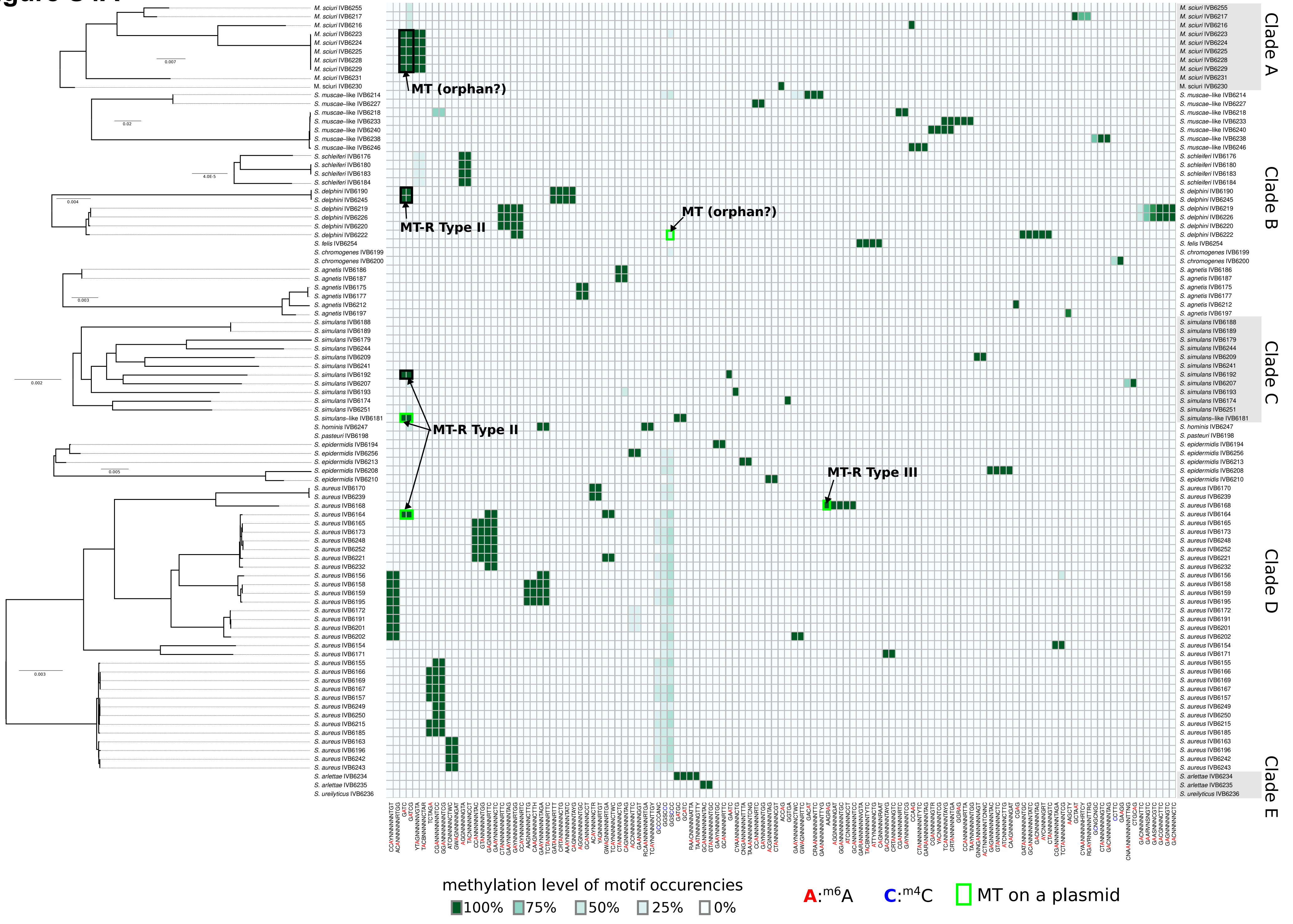

Clade A
Clade B
Clade C
Clade D
Clade E
MT (orphan?)
MT-R Type II
MT-R Type III
methylation level of motif occurencies
100%
75%
50%
25%
0%
A:m6A
C:m4C
MT on a plasmid

Figure S4B

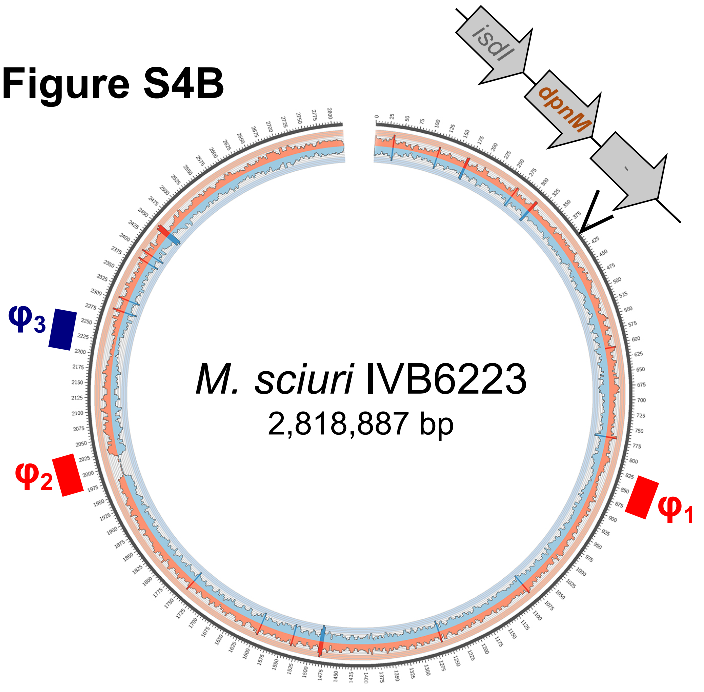

Figure S4C

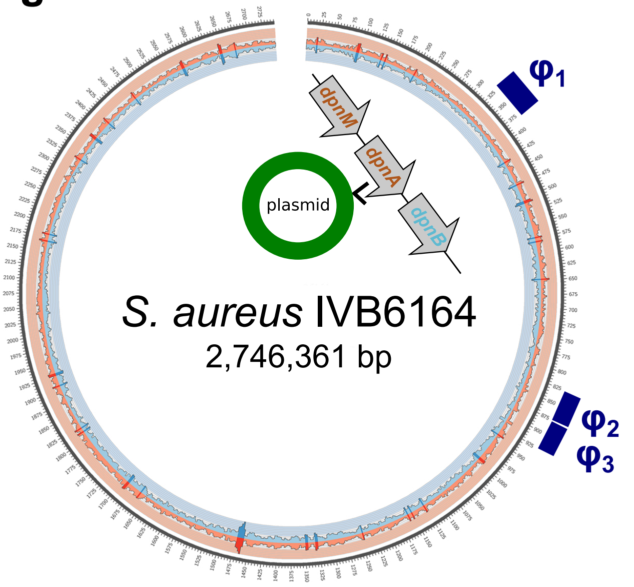

Figure S4D

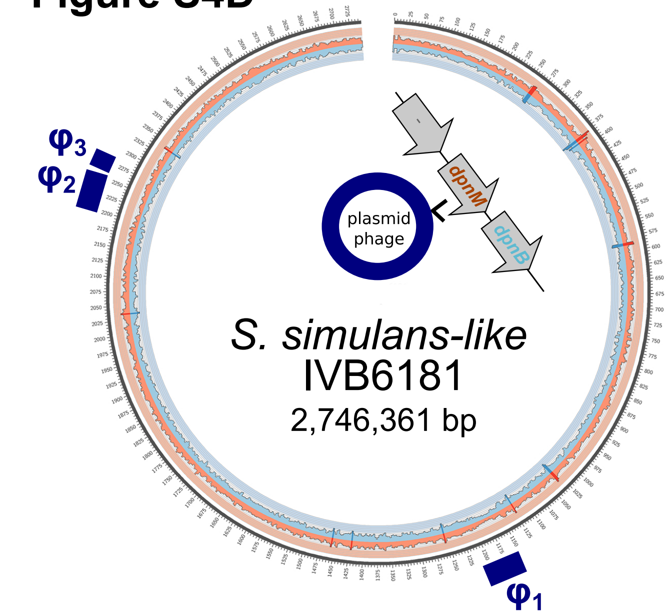

Figure S4E

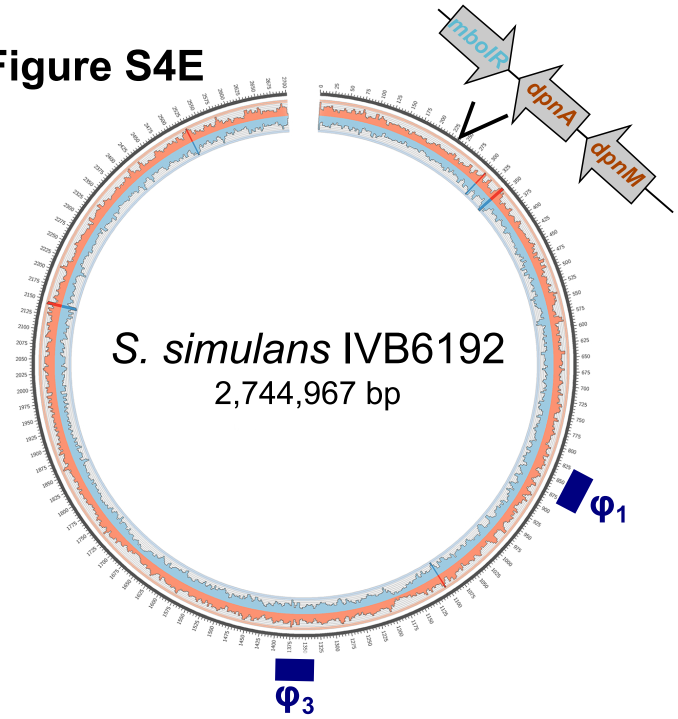

Figure S4F

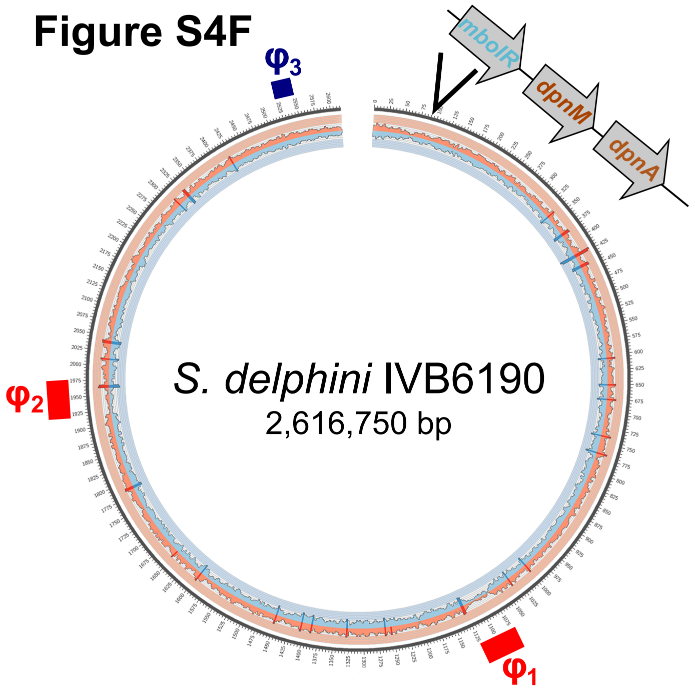

Figure S4G

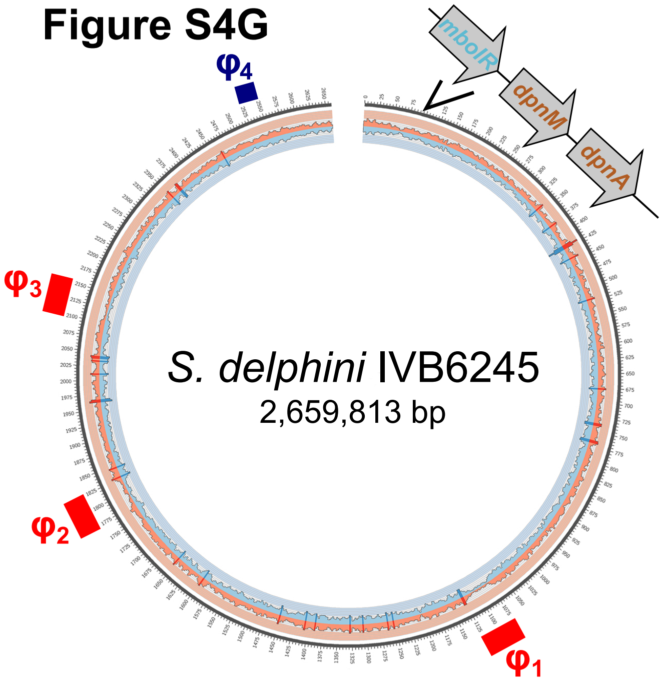

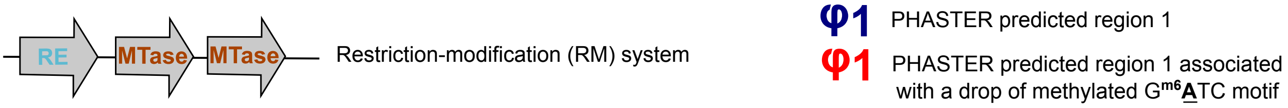

Figure S4. DNA methylation profiling and selective mapping of methylated G$^{m6}\underline{A}$TC motif in East African *Staphylococcaceae*. (A) PacBio DNA methylation profiling by quantification of ^{m6}A and ^{m4}C methylations in *Staphylococcaceae* strains (N=91). Motifs with ^{m6}A and ^{m4}C base modifications were identified (x-axis) in the sequenced genomes including plasmids and set in relation to the core genome based phylotree (y-axis). The methylation level of motifs found is indicated in % using a colour code. MT-R corresponds to the Methylase-Restriction enzyme system. MT-R Type II systems refer to the most common MT-R where MT and R act independently and compete for the same palindromic motif. MT-R Type III systems form heterodimers with the MTs methylating a single DNA strand. **(B-G) Genome mapping of the methylated G$^{m6}\underline{A}$TC motif.** For each genome where G$^{m6}\underline{A}$TC motif was identified, the corresponding draft genomic sequence and the G$^{m6}\underline{A}$TC motif coordinates were uploaded to PACific biosciences Methylation Analyzer (PACMAN) result (https://bugfri.unibe.ch). The PHASTER predicted prophage sequences were overlaid on each plot. The putative restriction-modification system involved in the G$\underline{A}$TC motif methylation or restriction was also placed.
